# Interface engineering breaks both stability and activity limits of RuO_2_ for sustainable water oxidation

**DOI:** 10.1038/s41467-022-33150-x

**Published:** 2022-09-16

**Authors:** Kun Du, Lifu Zhang, Jieqiong Shan, Jiaxin Guo, Jing Mao, Chueh-Cheng Yang, Chia-Hsin Wang, Zhenpeng Hu, Tao Ling

**Affiliations:** 1 Key Laboratory for Advanced Ceramics and Machining Technology of Ministry of Education, Institute of New-Energy, School of Materials Science and Engineering, Tianjin University, Tianjin, 300072 China; 2 School of Physics, Nankai University, Tianjin, 300071 China; 3 School of Chemical Engineering and Advanced Materials, The University of Adelaide, Adelaide, SA 5005 Australia; 4 National Synchrotron Radiation Research Center, Hsinchu, 30076 Taiwan, ROC; 5 Department of Materials Science and Engineering, National Yang Ming Chiao Tung University, Hsinchu, 30010 Taiwan, ROC

**Keywords:** Electrocatalysis, Energy, Electrocatalysis

## Abstract

Designing catalytic materials with enhanced stability and activity is crucial for sustainable electrochemical energy technologies. RuO_2_ is the most active material for oxygen evolution reaction (OER) in electrolysers aiming at producing ‘green’ hydrogen, however it encounters critical electrochemical oxidation and dissolution issues during reaction. It remains a grand challenge to achieve stable and active RuO_2_ electrocatalyst as the current strategies usually enhance one of the two properties at the expense of the other. Here, we report breaking the stability and activity limits of RuO_2_ in neutral and alkaline environments by constructing a RuO_2_/CoO_*x*_ interface. We demonstrate that RuO_2_ can be greatly stabilized on the CoO_*x*_ substrate to exceed the Pourbaix stability limit of bulk RuO_2_. This is realized by the preferential oxidation of CoO_*x*_ during OER and the electron gain of RuO_2_ through the interface. Besides, a highly active Ru/Co dual-atom site can be generated around the RuO_2_/CoO_*x*_ interface to synergistically adsorb the oxygen intermediates, leading to a favourable reaction path. The as-designed RuO_2_/CoO_*x*_ catalyst provides an avenue to achieve stable and active materials for sustainable electrochemical energy technologies.

## Abstract (web summary)

RuO_2_ encounters critical electrochemical dissolution issues during oxygen evolution reaction and it remains a grand challenge to achieve stable and active RuO_2_ electrocatalyst. Here, the authors report breaking stability and activity limits of RuO_2_ by constructing a RuO_2_/CoO_*x*_ interface.

## Introduction

The practical application of water electrolyser in the generation of sustainable green hydrogen energy^1–3^ calls for the development of stable and active electrocatalysts. So far, RuO_2_ is the most active electrocatalyst for anodic oxygen evolution reaction (OER) in water electrolysis^4–9^. Unfortunately, as indicated by Pourbaix diagram^10–12^, RuO_2_ is thermodynamically unstable under OER conditions over the entire pH range. This has been verified by extensive theoretical and experimental investigations^4,13–15^, which demonstrate that the proceeding of OER is accompanied by the transformation of stable Ru^4+^ to unstable Ru^n>4+^, resulting in the gradual dissolution and deactivation of the catalyst. Common strategies of improving the stability of RuO_2_ include mixing RuO_2_ with a more corrosion resistant material in the synthetic procedure^6,16–19^ and controlling the dispersion of RuO_2_ to avoid direct contact with the electrolyte^20^. In these cases, however, the stability of Ru-based catalysts is generally enhanced at the expense of its activity, leading to a seesaw relation between stability and activity^14,21–26^. It is necessary to develop new strategy to achieve both enhanced stability and activity for Ru-based catalysts.

To substantially enhance the stability of RuO_2_ catalysts under OER conditions, we identify that the key is to suppress the electrochemical corrosion of Ru species. There is a classic fashion of using a sacrifice component to protect the target material. For example, in the well-known zinc-plated steel^27^, the more reactive zinc is preferentially oxidized to form a dense oxide film over the steel, preventing the further oxidation of zinc and the corrosion of steel. Inspired by this, we assumed that implementing a proper material with RuO_2_ to form a stable interface can be a promising strategy to stabilize RuO_2_ catalyst. On the other hand, previous works of Nørskov et al.^14,15^ have suggested that the ‘stable’ RuO_2_ exhibits unsatisfactory catalytic activity due to the lack of unstable high-valence Ru^n>4+^ species. Regarding this, the construction of an interface may create new active sites^28^ to break the activity limit of ‘stable’ RuO_2_. Moreover, the interface construction may use some cost-effective materials to reduce the use of precious metal Ru and achieve sustainable water electrolysis.

Herein, we report constructing a RuO_2_/CoO_*x*_ hybrid catalyst to break the stability-activity seesaw relation on RuO_2_ catalyst. Combining theoretical calculations, in situ X-ray photoelectron spectroscopy (XPS) with in situ UV-visible (UV–Vis) absorption spectroscopy, we demonstrate that the stability of the new RuO_2_/CoO_*x*_ hybrid significantly exceeds the Pourbaix limits of bulk RuO_2_. This is ascribed to the sacrificing oxidation of CoO_*x*_ and interfacial electronic effects, which stabilized RuO_2_ by decreasing driving force for RuO_2_ dissolution and enriching electrons on RuO_2_. In addition, as verified by kinetic isotope effect (KIE), in situ infrared reflection (IR) measurements and theoretical calculations, the construction of interface creates highly active Ru/Co dual-atom sites around the RuO_2_/CoO_*x*_ interface, which synergistically absorb the key oxygen intermediates during OER to optimize the reaction thermodynamics and kinetics. Therefore, the RuO_2_/CoO_*x*_ catalyst achieves superior high OER activities under neutral and alkaline conditions accompanied by excellent long-term stability.

## Results

### Stabilization of RuO_2_ on CoO_*x*_ support

**Fig. 1:**
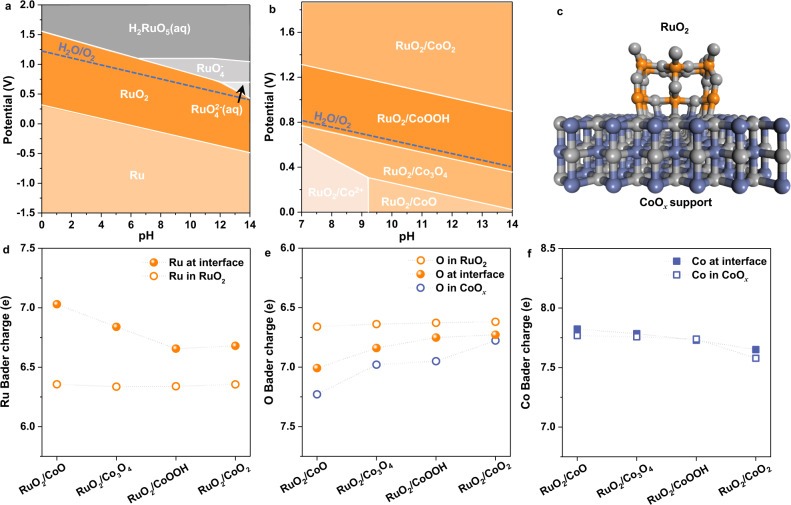
Investigation on stability of RuO2 on CoO*x* support. **a**, **b** Calculated Pourbaix diagrams of RuO_2_ and RuO_2_/CoO_*x*_, respectively. Ion concentrations are 10^−6^ M. The potentials in **a** and **b** are referenced to standard hydrogen electrode (SHE). **c** Schematic diagram of the interfacial structure of RuO_2_/CoO_*x*_. **d–f** Bader charges of the interfacial Ru, O and Co ions and their counterparts in RuO_2_/CoO, RuO_2_/Co_3_O_4_, RuO_2_/CoOOH and RuO_2_/CoO_2_, respectively.

According to our calculated Pourbaix diagram of RuO_2_ (Fig. 1a), RuO_2_ undergoes oxidation in the OER potential range, forming high-valence Ru^n>4+^ ions that dissolve in the electrolyte^4,13,29^. We assume that depositing RuO_2_ on an appropriate support that can be preferentially oxidized represents a rational strategy to protect RuO_2_ from dissolution in harsh electrochemical oxidation. To test this hypothesis, CoO_*x*_ was selected as the support material, which is easily oxidized under the anodic potential in the OER range (Supplementary Fig. 1). The calculated Pourbaix diagram of RuO_2_/CoO_*x*_ (Supplementary Note 1) in near-neutral and alkaline environments is shown in Fig. 1b. As expected, the CoO_*x*_ support is gradually oxidized from CoO to Co_3_O_4_, CoOOH and eventually CoO_2_ with the increase of anodic potential. Hereafter, CoO_*x*_ repents these cobalt oxides for simplicity. Significantly, RuO_2_ can construct stable interfaces with the oxidation products of CoO_*x*_ (CoO, Co_3_O_4_, CoOOH, and CoO_2_) within the entire OER potential range (Fig. 1b, c). Besides, stable Ru–O–Co chemical bond can be formed at the RuO_2_/CoO_*x*_ interface (Fig. 1c and Supplementary Fig. 2), which enables the hybrid to gain considerable energy from constructing the interface (Supplementary Fig. 2). This undoubtedly lowers the energy of the hybrid system and decreases the driving force for RuO_2_ dissolution, thus stabilizing RuO_2_ in the hybrid catalyst.

To further understand the interfacial effect on stabilizing RuO_2_, Bader charge analysis was performed on four RuO_2_/CoO_*x*_ catalysts, i.e., RuO_2_/CoO, RuO_2_/Co_3_O_4_, RuO_2_/CoOOH, and RuO_2_/CoO_2_. As shown in Fig. 1d–f, the changes in the charges of Ru, O and Co ions at the interface relative to those in their corresponding bulk materials show a similar trend among the four catalysts. Taking RuO_2_/CoOOH as an example, the average charge of Ru ions away from the interface in RuO_2_ is ~6.3 *e*, which increases to 6.7 *e* at the interface (Fig. 1d), indicating the enrichment of electrons on the interfacial Ru ions. Similarly, the average charge of O ions in the bulk RuO_2_ is ~6.6 *e*, which increases to ~6.7 *e* at the interface, and further increases to ~7.0 *e* in the bulk CoOOH (Fig. 1e). Note that the Co charge at the interface is almost identical to that in the bulk CoOOH (Fig. 1f). These results indicate that O ions in the hybrids play a key role in the electron enrichment in interfacial Ru ions. This is due to the different metal-oxygen hybridizations in RuO_2_ and CoOOH, resulting in different O charges in these two materials. That is, the O ions connecting with Co ions own more electrons compared with those connecting with Ru ions. Once Ru–O–Co bond is formed at the RuO_2_/CoO_*x*_ interface, the electron-rich O ions connecting with Co ions contribute electrons to the nearby Ru ions through metal-oxygen re-hybridization, thus enriching electrons in the interfacial Ru ions.

### Synthesis of RuO_2_/CoO_*x*_ hybrid catalyst

**Fig. 2:**
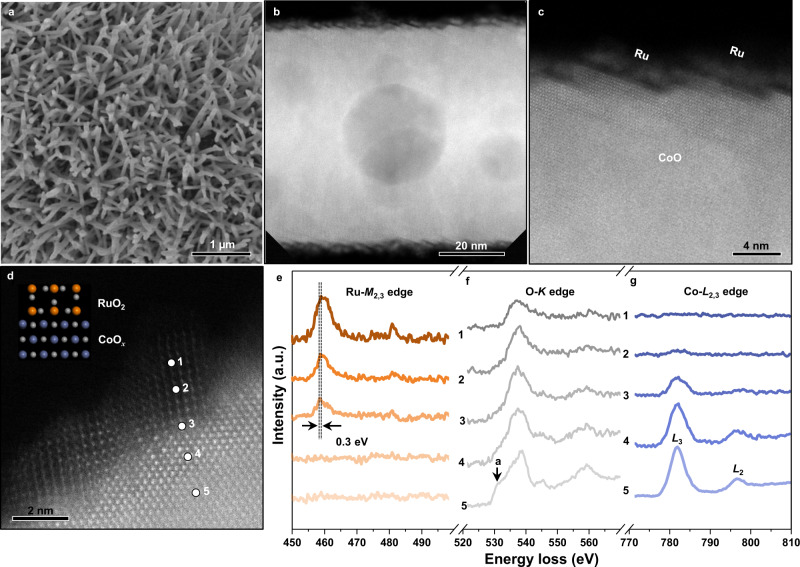
Synthesis of RuO2/CoO*x* hybrid catalyst. **a** Low-magnification SEM image of Ru/CoO. **b**, **c** Low- and high-magnification HADDF-STEM images of Ru/CoO, respectively. **d**, Atomic resolution HADDF-STEM image of RuO_2_/CoO_*x*_, with the inset showing the atomic model of RuO_2_/CoO_*x*_. **e–g** EELS spectra of Ru-*M*_2,3_, O-*K*, and Co-*L*_2,3_ edge across the interface from point 1 to point 5 in **d**, respectively.

Guided by the above theoretical findings, RuO_2_/CoO_*x*_ hybrid catalyst was fabricated by depositing Ru nanoparticles on CoO nanorods (Fig. 2a), followed by an electrochemical oxidization process (Supplementary Figs. 3–8). As shown in Fig. 2b, c, the CoO nanorods possess faceted surface with prefabricated nanoscale roughness to uniformly load Ru nanoparticles. The Ru nanoparticles form a fish scale-like single-layer with a thickness of 2 nm on the surface of CoO nanorods (Supplementary Fig. 5). Subsequent electrochemical oxidation resulted in in situ conversion of Ru to RuO_2_ on CoO_*x*_ nanorods (Supplementary Figs. 7 and 8). This method features the epitaxial growth of RuO_2_ on CoO_*x*_ nanorods (Fig. 2d), providing a structural basis for strong interfacial geometric and electronic interaction between RuO_2_ and CoO_*x*_. The as-formed interface was closely inspected by sub-ångstrom resolution aberration corrected high-angle annular dark-field scanning transmission electron microscopy (HAADF-STEM, Fig. 2d and Supplementary Fig. 9), showing an atomic-level tight connection of Ru, O and Co atoms at the interface. This finding was supported by the Fourier transform extended X-ray absorption fine structure (FT-EXAFS) of RuO_2_/CoO_*x*_ (Supplementary Fig. 10).

Electron energy-loss spectroscopy (EELS) at Ru-*M*_2,3_, O-*K*, and Co-*L*_2,3_ absorption edges was performed to investigate charge changes of Ru, O and Co ions across the interface (from point 1 to point 5) in Fig. 2d. As illustrated in Fig. 2e, the collected Ru-*M*_2,3_ spectrum at the interface (point 3) shifts 0.3 eV toward the low energy loss direction with respective to that of RuO_2_ (point 1), indicating a decreased Ru valence at the interface. For O-*K* edge spectra (Fig. 2f), the curves show obvious shape change from RuO_2_-like (point 1) to CoO_*x*_-like (point 5). In particular, the characteristic peak ‘a’ collected in CoO_*x*_ gradually weakens towards the interface until disappears in RuO_2_. This reflects different electronic properties of O atoms connecting with Ru and Co atoms, respectively, and re-hybridization of O atoms at the interface caused by simultaneous connection with Ru and Co atoms. Notably, no noticeable peak shift is observed in the collected Co-*L*_2,3_ spectra (Fig. 2g and Supplementary Fig. 11). These experimental results well support the calculated evident charge change of O ions from CoO_*x*_ to RuO_2_ via the interface (Fig. 1e), while no significant Co charge change from bulk CoO_*x*_ to the interface (Fig. 1f). This indicates that O ions play a decisive role in the reduction of Ru valence through the electronic interaction among Ru, O and Co atoms at the interface. We note that the enrichment of Ru charge at the interface will affect the distribution of Ru charge in the bulk and on the surface through continuous Ru–O bonds.

### Stability evaluation of RuO_2_/CoO_*x*_ in OER

**Fig. 3:**
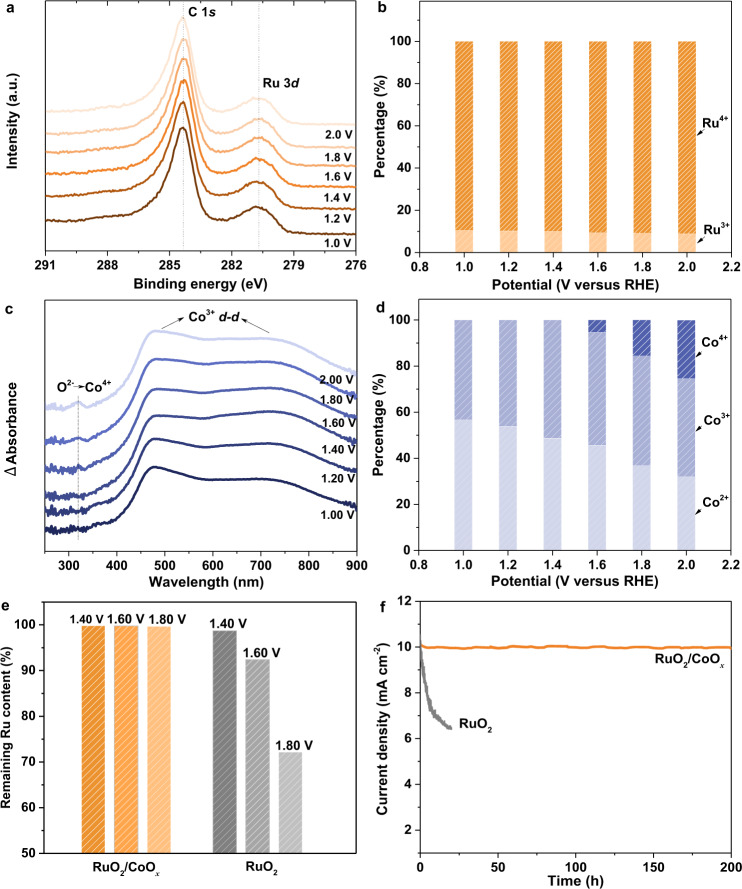
Investigation on in situ stability of RuO2/CoO*x* hybrid catalyst during OER. **a**, **b** In situ Ru 3*d* XPS spectra recorded at applied potential during 1.00–2.00 V_RHE_ and corresponding Ru^3+^ and Ru^4+^ content ratios, respectively. **c** In situ UV–Vis spectra recorded at applied potential during 1.00–2.00 V_RHE_. The spectrum at each potential was collected by subtracting the spectrum of pristine RuO_2_/CoO_*x*_. **d** Co^2+^, Co^3+^ and Co^4+^ content ratios in RuO_2_/CoO_*x*_ under different applied potentials based on EPR analysis. **e** Retention ratios of Ru in RuO_2_/CoO_*x*_ and RuO_2_ after continuously tested at different potentials for 20 h. **f** Potentiostatic tests of RuO_2_/CoO_*x*_ (at 1.47 V_RHE_) and RuO_2_ (at 1.59 V_RHE_) for an initial current density of 10 mA cm^−2^.

Afterwards, the stability of RuO_2_/CoO_*x*_ hybrid catalyst during OER in neutral environment was monitored by in situ XPS (Supplementary Figs. 12 and 13). Significantly, the Ru 3*d* XPS peak at 280.9 eV exhibits negligible changes with the applied potential increased from 1.0 to 2.0 V versus reversible hydrogen electrode (RHE) (Fig. 3a). Detailed quantitative analysis shows the co-existence of Ru^3+^ and Ru^4+^ species with almost identical percentages from 1.0 to 2.0 V_RHE_ (Fig. 3b and Supplementary Fig. 14). Surprisingly, even at 2.0 V_RHE_, there is still 9% of Ru^3+^ remaining in the RuO_2_/CoO_*x*_ hybrid. Considering that the average particle size of RuO_2_ is ~2 nm, the theoretical proportion of interfacial Ru atoms to total Ru atoms should be about 15% (Supplementary Note 2 and Supplementary Fig. 15). This value is in agreement with the percentage of Ru^3+^ species as demonstrated by the in situ XPS results (Fig. 3b), indicating the critical role of the constructed interface in stabilizing RuO_2_ in the hybrid.

Moreover, although the Ru valence state of RuO_2_/CoO_*x*_ hybrid did not exceed 4+ in the studied potential range, the Co valence state increased significantly during OER as evidenced by in situ UV–Vis spectroscopy characterization and quantitative electron paramagnetic resonance (EPR) analysis. It was demonstrated that as the anodic potential increased, the Co ions in the hybrid catalyst underwent gradual oxidation from Co^2+^ to Co^3+^ and Co^4+^ without dissolution (Fig. 3c, d, Supplementary Fig. 16 and Supplementary Table 1). This is consistent with the calculated Pourbaix diagram of the hybrid catalyst (Fig. 1b) and verified our hypothesis that the support CoO_*x*_ was preferentially oxidized to protect RuO_2_.

The above in situ spectroscopic results were supported by the experimentally observed remarkable stability of RuO_2_/CoO_*x*_ during OER. As shown in Fig. 3e, Supplementary Figs. 17 and 18 and Supplementary Table 1, after 20 h continuous stability test at the potential as high as 1.80 V_RHE_, the content of Ru element in the hybrid catalyst was still close to 100%. Significantly, the RuO_2_/CoO_*x*_ catalyst works stably at a constant current density of 10 mA cm^–2^ for more than 200 h (Fig. 3f), and affords an excellent dynamic stability with varied current density from 10 to 100 mA cm^−2^ (Supplementary Fig. 19). In sharp contrast, the pristine RuO_2_ (deposited on carbon black, Supplementary Figs. 20 and 21) encountered severe catalyst dissolution and performance degradation (Fig. 3f and Supplementary Fig. 22), which agrees well with the literature^4,13,29^. Additionally, the RuO_2_/CoO_*x*_ also demonstrated excellent stability in alkaline environment (Supplementary Figs. 23, 24 and Supplementary Table 2).

### OER activity and rate-determining step of RuO_2_/CoO_*x*_

**Fig. 4:**
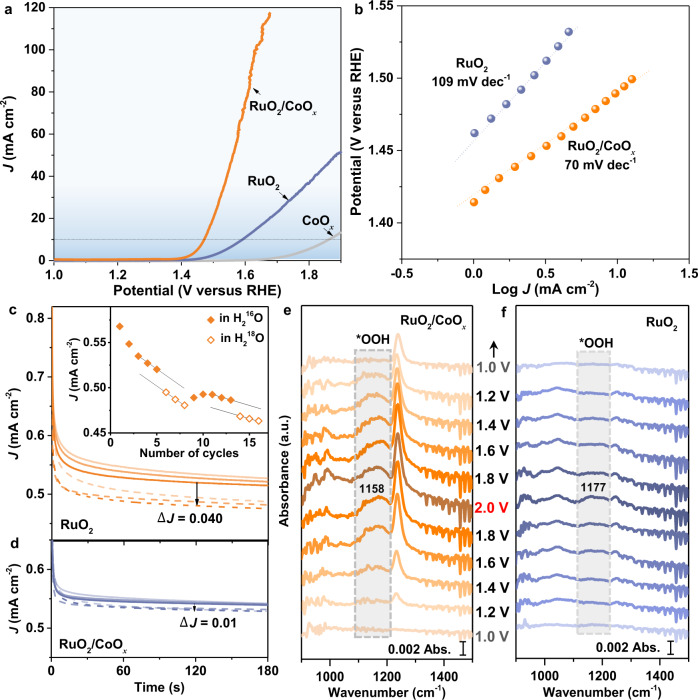
OER performance and RDS of RuO2/CoO*x* hybrid catalyst. **a**, **b** OER polarization curves of RuO_2_/CoO_*x*_, RuO_2_ (deposited on carbon black) and CoO_*x*_, and corresponding Tafel curves, respectively. **c**, **d** Current density (*J*)-time curves of RuO_2_ and RuO_2_/CoO_*x*_ with multiple cycles in either H_2_^16^O (solid lines, closed triangles) or H_2_^18^O (dashed lines, open triangles), respectively. The inset in **c** represents the average current density (*J*_average_) during the last 2 minutes for each cycle. **e**, **f** In situ surface-enhanced IR spectra of RuO_2_/CoO_*x*_ and RuO_2_ at different potentials. The potentials were referenced to RHE. Note that the catalysts here were tested in neutral electrolyte and the RuO_2_ loading on CoO_*x*_ is 10 µg on per cm^2^ electrode.

Under the incentive of the high stability, we evaluated the OER activity of the RuO_2_/CoO_*x*_ hybrid catalyst with a RuO_2_ mass loading of 10 µg on per cm^2^ electrode (Supplementary Table 3, Supplementary Figs. 25 and 26). Note that RuO_2_ (Supplementary Fig. 27 and Supplementary Table 4) and CoO_*x*_ catalysts were measured as control samples. As shown in Fig. 4a and Supplementary Fig. 28, the RuO_2_/CoO_*x*_ exhibits a much higher OER activity than RuO_2_ and CoO_*x*_ in neutral electrolyte, affording an ultra-low overpotential of 0.24 V to drive an OER current density of 10 mA cm^–2^. Besides, the current density of RuO_2_/CoO_*x*_ can achieve 400 mA cm^−2^ at 1.92 V_RHE_ when the mass of RuO_2_/CoO_*x*_ catalyst is increased to 1.5 mg cm ^−2^ on nickel foam (Supplementary Fig. 29). Impressively, the RuO_2_/CoO_*x*_ is amongst the most active OER catalysts reported so far under neutral conditions (Supplementary Table 5). Moreover, the turnover frequency (TOF) of the RuO_2_/CoO_*x*_ was estimated by normalizing the O_2_ generation rate to the total number of Ru ions on CoO_*x*_ support (Supplementary Note 3). At an overpotential of 400 mV, the RuO_2_/CoO_*x*_ delivers a high TOF of 3.61 s^–1^, representing a 10-time enhancement in comparison with the optimum value reported previously on Ru-based catalyst (RuIrCaO_*x*_^30^, 0.36 s^–1^). Moreover, the RuO_2_/CoO_*x*_ achieves a high OER Faradaic efficiency of ~98% at 10 mA cm^−2^ (Supplementary Fig. 30).

To reveal the activity origin of the RuO_2_/CoO_*x*_, we explored the rate-determining step (RDS) of OER by Tafel plots. As illustrated in Fig. 4b, the RuO_2_/CoO_*x*_ shows a significantly decreased Tafel slope (70 mV dec^–1^) compared with the RuO_2_ (109 mV dec^–1^), indicating the possible different RDSs in these two catalysts. ^18^O/^16^O isotope effect^31^ was then employed in both catalysts to probe the O–O bond formation, which is generally considered as the RDS in OER^32,33^. As shown in Fig. 4c, there is an obvious decrease in the OER current density on the RuO_2_ catalyst when the electrolyte was changed from H_2_^16^O to H_2_^18^O, and the KIE value of the O–O bond formation step (KIE_O-O_) is estimated as 1.03 (Supplementary Fig. 31). Since the KIE_O-O_ value falls within the range between 1.01 and 1.04^34,35^, the O–O bond formation step can be confirmed to be the RDS of the RuO_2_. In contrast, the negligible ∆*J* between H_2_^16^O and H_2_^18^O for the RuO_2_/CoO_*x*_ demonstrates that O–O bond formation is not the RDS (Fig. 4d). This finding is further supported by the in situ IR spectroscopy characterization (Fig. 4e, Supplementary Fig. 32 and Supplementary Table 6), which shows a more pronounced *OOH band of RuO_2_/CoO_*x*_ in comparison with that of RuO_2_ (Fig. 4f). These results suggest that the RuO_2_/CoO_*x*_ exhibits a different RDS compared with the pristine RuO_2_ as we will discuss in detail later.

Furthermore, we demonstrate that the RuO_2_/CoO_*x*_ hybrid catalyst delivers a superior high OER performance in alkaline environment, permitting it a promising candidate for highly efficient OER electrocatalysts in a wide pH range (Supplementary Fig. 33 and Supplementary Table 7).

### Origin of enhanced OER activity on RuO_2_/CoO_*x*_

**Fig. 5:**
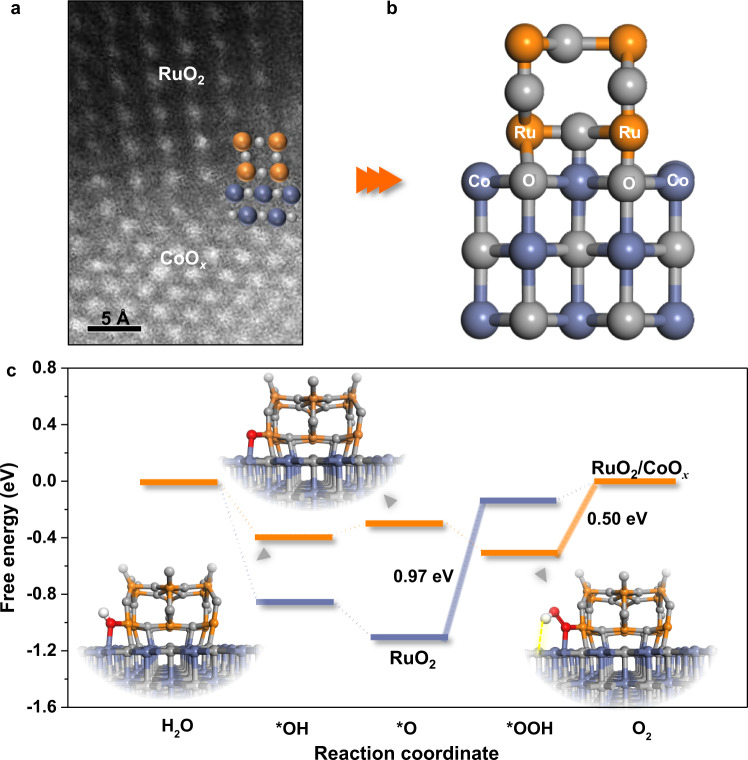
Theoretical study on origin of enhanced activity on RuO2/CoO*x*. **a** HADDF-STEM characterization of RuO_2_/CoO_*x*_ interface exposed active sites. **b** Schematic diagram of the structure model of RuO_2_/CoO_*x*_ hybrid catalyst. **c** Calculated OER free energy diagrams for RuO_2_ and RuO_2_/CoO_*x*_, with the inset showing the computationally-optimized geometric structures of *OH, *O and *OOH intermediates co-adsorbed on Ru/Co dual-atom site exposed around the RuO_2_/CoO_*x*_ interface.

A key question remains how the RuO_2_/CoO_*x*_ interface significantly boosts the OER activity of RuO_2_. To shed light on this, density functional theory (DFT) calculations were performed. In particular, HADDF-STEM imaging (Fig. 5a) shows that Ru/Co dual-atom sites were exposed around the RuO_2_/CoO_*x*_ interface after treating the hybrid at the OER onset potential (~1.40 V_RHE_). Accordingly, the computational model was constructed (Fig. 5b). It was found that the exposed Ru/Co dual-atom site around the interface is the most active site for OER (Supplementary Figs. 34–36); the oxygen intermediates, i.e., *OH, *O and *OOH, tend to be co-adsorbed at the Ru/Co dual-atom site to form a stable nearly quadrilateral structure (Fig. 5c, inset).

Significantly, the triatomic *OOH bents downward and the H atom forms a hydrogen bond with the surface O in the CoO_*x*_ to construct a unique ${}^{\ast }{{{{{\rm{O}}}}}}{{{{{\rm{O}}}}}}{-}{{{{{\rm{H}}}}}}\cdots {{{{{\rm{O}}}}}}$ adsorption configuration. Due to the electrostatic attraction of O atom in the CoO_*x*_, the O–H bond length in the formed *OOH increases compared with that on the pristine RuO_2_ (Supplementary Fig. 35). According to previous work^36^, when the intermolecular hydrogen bond stretches the bond in the probe molecule, it will lead to a shift of the stretching vibrational frequency of the probe groups toward the low wavenumber direction in IR spectra. Relative shift of *OOH bands is observed in the in situ IR spectra of the RuO_2_/CoO_*x*_ compared with those of RuO_2_ (Fig. 4e, f), verifying the adsorption configuration of ${}^{\ast }{{{{{\rm{O}}}}}}{{{{{\rm{O}}}}}}{-}{{{{{\rm{H}}}}}}\cdots {{{{{\rm{O}}}}}}$, which facilitates the stabilization of *OOH at the Ru/Co dual-atom site around the interface (inset of Fig. 5c and Supplementary Fig. 35).

Note that *OOH is a key intermediate during OER, which exhibits a high formation barrier and restricts the OER activity of catalysts^32,33^. The calculated Gibbs free energy for *OOH formation (ΔG_*OOH_) on the RuO_2_ is as high as 1.12 eV (Fig. 5c and Supplementary Fig. 35). Notably, this calculated value of ΔG_*OOH_ is consistent with the result reported by Nørskov et al. and other researchers^15,24^, indicating an inferior OER activity of ‘stable’ RuO_2_ with the absence of the generated high-valent Ru^n>4+^ species during OER^4,13,29^. As expected, for the RuO_2_/CoO_*x*_, the *OOH formation is greatly facilitated at the Ru/Co dual-atom site around the interface. More importantly, this shifts the RDS of RuO_2_/CoO_*x*_ to the subsequent step of *OOH formation – that is, desorption of *O_2_ (Supplementary Note 4), which demonstrates a significantly decreased energy injection of 0.50 eV (Fig. 5c). This exciting finding agrees well with the KIE and in situ IR results (Fig. 4c–f). Therefore, our well-consistent experiments and calculations confirm that the artificially constructed RuO_2_/CoO_*x*_ hybrid catalyst successfully breaks the OER activity limit of ‘stable’ RuO_2_ by changing the RDS of OER through exposing the highly active Ru/Co dual-atom sites around the RuO_2_/CoO_*x*_ interface (Supplementary Note 5 and Supplementary Figs. 37–39).

## Discussion

In summary, we constructed the RuO_2_/CoO_*x*_ hybrid catalyst to break the stability and activity limits of RuO_2_ by decoupling its stability-activity relation. Specifically, the sacrificial oxidization of CoO_*x*_ and the electron interaction among the face-to-face Ru–O–Co interfacial atoms enhance the stability, while the Ru/Co dual-atom site exposed around the interface is responsible for the improved activity. With such unique electronic and geometric effects generated by the RuO_2_/CoO_*x*_ interface, we solved the critical issues of RuO_2_ under OER conditions and achieved high stability and excellent activity. Our work provides an atomic scale understanding of employing interfacial effect to simultaneously enhance the stability and activity of RuO_2_. We believe that under the guideline built by the RuO_2_/CoO_*x*_ interface, the activity and stability issues of RuO_2_ in acidic environments can also be fundamentally solved by selecting appropriate support materials. We expect that this work will also contribute to future research on other renewable energy technologies coupled with OER in neutral environments, such as reduction of carbon dioxide to multi-carbon fuels.

## Methods

### Synthesis of RuO_2_/CoO_*x*_ and RuO_2_ catalysts

RuO_2_/CoO_*x*_ catalyst was synthesized by in situ electrochemical transformation method with Ru/CoO as the starting material. Briefly, CoO nanorod arrays were first fabricated on carbon fiber paper or fluorine-doped tin oxide (FTO) substrates by cation exchange methodology^37,38^. Afterwards, ruthenium precursor solution was prepared by dissolve RuCl_3_ in ethanol/water (V_ethanol_/V_water_ = 1:1) to achieve a 30 mM RuCl_3_ solution. Then, CoO nanorods were immersed in 40 mL of ultrapure water, and an appropriate amount of ruthenium precursor solution was added, aged for 6 h, dried at room temperature, and finally heated by N_2_ flow at 400, 500 and 550 °C for 0.5 h to obtain RuO_2_ with average particle sizes of 2, 3 and 4 nm, respectively (Supplementary Fig. 38). Note that the Ru loading mass on CoO nanorods can be easily controlled by tuning the adding volumes of ruthenium precursor solution. Finally, the obtained Ru/CoO nanorods were electrochemically oxidized by scanning cyclic voltammetry between 0.80~1.50 V_RHE_ to attain RuO_2_/CoO_*x*_ catalysts (Supplementary Fig. 8). The loading mass of RuO_2_ on CoO_*x*_ after optimization is 10 µg on per cm^2^ electrode (Supplementary Fig. 26 and Supplementary Table 3). For the synthesis of RuO_2_ reference catalyst, a similar method was applied using carbon black as the support material. The loading mass of RuO_2_ on carbon black after optimization is 84 µg on per cm^2^ electrode (Supplementary Fig. 27 and Supplementary Table 4). RuO_2_ with this loading mass was characterized in Figs. 3 and 4 as reference sample.

### Materials characterization

Scanning electron microscopic (SEM) and transmission electron microscopic (TEM) images were performed on a Hitachi S-4800 SEM and a JEOL 2100 TEM, respectively. HAADF-STEM images were collected on a JEOL ARM200F microscope with a STEM aberration corrector operated at 200 kV. The convergent semi angle and collection angle were 21.5 and 200 mrad, respectively. EELS spectra were collected using a Titan Themis Cubed G2 60-300 operated at 200 kV. EPR measurements were carried out on a JEOL JES-FA200. The inductively coupled plasma mass spectrometry (ICP-MS) measurements were performed on an Agilent 7700x. X-ray diffraction (XRD) characterization was carried out on a Bruker D8 Advance diffractometer with Cu Kα radiation. The X-ray absorption fine structure spectra of Ru *K*-edge were performed at 4B9A beamline in Beijing Synchrotron Radiation Facility (BSRF). The storage rings of BSRF was operated at 2.5 GeV with a stable current of 400 mA. The OER Faradaic efficiency of RuO_2_/CoO_*x*_ was measured by a gas chromatograph (GC-2014, Shimadzu, Japan) equipped with a thermal conductivity cell detector.

### In situ spectroscopic characterizations

In situ XPS spectra were measured by ambient pressure XPS end station equipped with a static electrochemical cell at NSRRC TLS BL24A (Supplementary Fig. 12a). The counter electrode was a Pt wire and the reference electrode was a Pt wire coated with Ag/AgCl paste. The working electrode was a carbon paper loaded with RuO_2_/CoO_*x*_ catalysts, which was cut into a circle with a diameter of 5.5 mm. During in situ XPS test, both the counter and reference electrodes were immersed in the electrolyte and sealed by a Nafion membrane and the carbon paper was sandwiched between the Nafion membrane and a Ta foil for electrical contact^39,40^ (Supplementary Fig. 12b). The analysis chamber pressure is around 0.3 mbar due to water diffusing onto the sample’s surface and evaporating into the chamber while in situ XPS spectra were measured.

In situ UV–vis spectroscopy was performed on a Hitachi U-3010 with a homemade photo-electrochemical cell, with catalysts fabricated in situ on a FTO substrate as the working electrode, a Pt wire as counter electrode and an Ag/AgCl electrode as the reference electrode.

In situ attenuated total reflectance surface-enhanced IR spectra were collected on a Fourier transform infrared spectrometer (Nicolet IS50, Thermo Fisher Scientific Co., Ltd) with a MCT detector and a Pike Technologies VeeMAX III ATR accessory. A catalyst ink was prepared by mixing 2 mg of catalyst investigated with 1 mL of ultrapure water and then deposited on an Au film coated Si prism. The Si prism, a Pt foil and an Ag/AgCl electrode were served as the working electrode, the counter electrode, and the reference electrode, respectively, in an H-type electrochemical cell, which was separated by a Nafion 115 membrane. All background curves were collected without applied potential in N_2_-saturated electrolyte, and all spectra were collected with a 4 cm^–1^ resolution.

### EPR tests

RuO_2_/CoO_*x*_ was treated at 1.00, 1.20, 1.40, 1.60, 1.80 and 2.00 V_RHE_ for 5 min, respectively, and then dried quickly by high-purity N_2_ (99.999%). Then, the treated catalysts were collected and transferred to an EPR tube under N_2_ atmosphere. Then, the tube was immediately frozen and stored at 77 K using liquid nitrogen. The EPR measurement was performed at a modulation amplitude of 0.8 mT, a modulation frequency of 100 kHz, a conversion time of 50 ms and a time constant of 50 ms. During test, the temperature was set at 70 K. Quantitative analysis was conducted by double integration after baseline correction^41^.

### KIE measurements

According to previous literature^31,34,35,42^, multicycles chronoamperometric tests were carried out in 1.0 M phosphate buffered saline (PBS) with H_2_^16^O and H_2_^18^O. The KIE value was estimated from the following equation:

1 $${{{{{\rm{KIE}}}}}}={J}_{{H}_{2}^{16}{{{{{\rm{O}}}}}}}\,/\,{J}_{{H}_{2}^{18}{{{{{\rm{O}}}}}}}$$

where ${J}_{{H}_{2}^{16}{{{{{\rm{O}}}}}}}$ and ${J}_{{H}_{2}^{18}{{{{{\rm{O}}}}}}}$ are the average current density in H_2_^16^O and H_2_^18^O, respectively. The average current density values of multicycles were linear fitted. The KIE value was estimated from the ratio of the two data points in the two fitted line in H_2_^16^O and H_2_^18^O (Supplementary Fig. 31).

### Electrochemical characterizations

The electrochemical performance of the catalysts in neutral (1.0 M PBS) and alkaline (1.0 M KOH) electrolytes was tested in a three-electrode system. A catalyst ink was prepared by ultrasonically dispersing 2 mg of catalyst, 2 mg of conductive carbon (Vulcan XC 72), 20 μL of 5 wt% Nafion solution and 20 μL of isopropanol in ultrapure water to achieve a catalyst concentration of 5 mg mL^–1^. 10 μL of as-prepared catalyst ink was then dropped onto a polished glassy carbon rotating electrode (5 mm in diameter, Pine Research Instrumentation) serving as the working electrode (Supplementary Tables 3 and 4). The counter electrode was a Pt wire and the reference electrode was a calomel electrode saturated in KCl. The electrochemical tests were performed in O_2_-saturated electrolyte with the working electrode rotating at a speed of 1600 rpm. All potentials were referenced to the RHE by using pure hydrogen calibration and corrected with 75% *IR* loss, and all polarization curves were obtained with a scan rate of 5 mV s^–1^.

### Computational methods

All spin-polarized DFT calculations were performed using Vienna *Ab* initio Simulation Package (VASP)^43–46^. The projector augmented wave (PAW) potentials^47^ and Perdew-Burke-Ernzerhof (PBE) exchange-correlation functional^48^ were adopted in the calculations with a plane wave kinetic energy cut-off of 400 eV. The energy converge criteria was set to be 10^–4^ eV, and the maximum force was converged to less than 0.05 eV Å^−1^ on each ion. An effective *U* parameter of 3.7 eV was applied for Co 3*d* states to describe well the electronic structure of CoO, Co_3_O_4_, CoOOH, and CoO_2_^38^. For the computational model of RuO_2_/CoO_*x*_, the optimized lattice constants are *a* = *b* = 18.10 Å, c = 26.40 Å; for RuO_2_/Co_3_O_4_, *a* = *b* = 16.80 Å, c = 27.43 Å; for RuO_2_/CoOOH, *a* = *b* = 17.30 Å, c = 32.07 Å; for RuO_2_/CoO_2_, *a* = *b* = 17.06 Å, c = 31.54 Å. *K*-spaces were sampled using a 1 × 1 × 1 grid. The free energy (Δ*G*) was computed from the following equation:

2 $$\varDelta G=\varDelta E+\varDelta {{{{{\rm{ZPE}}}}}}-T\varDelta S-eU$$

where Δ*E* is the energy difference of a given reaction, ΔZPE is the zero-point energy correction, Δ*S* is the vibrational entropy change at a given temperature *T*, *e* is the elementary charge, and *U* is the electrode potential.

## Supplementary information

Supplementary Information

Peer review file

## Data Availability

The data that support the findings of this study are available from the corresponding author on reasonable request.
