# Publisher Correction: Interface engineering breaks both stability and activity limits of RuO_2_ for sustainable water oxidation

**DOI:** 10.1038/s41467-022-33557-6

**Published:** 2022-09-29

**Authors:** Kun Du, Lifu Zhang, Jieqiong Shan, Jiaxin Guo, Jing Mao, Chueh-Cheng Yang, Chia-Hsin Wang, Zhenpeng Hu, Tao Ling

**Affiliations:** 1 Key Laboratory for Advanced Ceramics and Machining Technology of Ministry of Education, Institute of New-Energy, School of Materials Science and Engineering, Tianjin University, Tianjin, 300072 China; 2 School of Physics, Nankai University, Tianjin, 300071 China; 3 School of Chemical Engineering and Advanced Materials, The University of Adelaide, Adelaide, SA 5005 Australia; 4 National Synchrotron Radiation Research Center, Hsinchu, 30076 Taiwan, ROC; 5 Department of Materials Science and Engineering, National Yang Ming Chiao Tung University, Hsinchu, 30010 Taiwan, ROC

**Keywords:** Electrocatalysis, Energy, Electrocatalysis

**Correction to**: *Nature Communications* 10.1038/s41467-022-33150-x, published online 16 September 2022

In this article Chia-Hsin Wang was incorrectly denoted as being one of the equally contributing authors. The original article has been corrected.

