# Supplementary material for: Interface engineering breaks both stability and activity limits of RuO2 for sustainable water oxidation
Source: Nat Commun. 2022 Sep 16;13:5448. doi: 10.1038/s41467-022-33150-x (PMC9481627; doi:10.1038/s41467-022-33150-x)
Supplement: Supplementary file 1 — Supplementary Information [file 41467_2022_33150_MOESM1_ESM.pdf]

Interface engineering breaks both stability and activity limits of RuO_2 for sustainable water oxidation

Kun Du[1,†], Lifu Zhang[2,†], Jieqiong Shan[3], Jiaxin Guo[1], Jing Mao[1], Cheuh-Cheng Yang[4,5], Chia-Hsin Wang[4,*], Zhenpeng Hu[2,*], Tao Ling[1,*]

[1] Key Laboratory for Advanced Ceramics and Machining Technology of Ministry of Education, Institute of New-Energy, School of Materials Science and Engineering, Tianjin University, Tianjin 300072, China.

[2] School of Physics, Nankai University, Tianjin 300071, China.

[3] School of Chemical Engineering and Advanced Materials, The University of Adelaide, Adelaide, SA 5005, Australia.

[4] National Synchrotron Radiation Research Center, Hsinchu, 30076, Taiwan.

[5] Department of Materials Science and Engineering, National Yang Ming Chiao Tung University, Hsinchu, 30010, Taiwan.

[†] These authors contributed equally to this work.

*Email: lingt04@tju.edu.cn; zphu@nankai.edu.cn; wang.ch@nsrrc.org.tw

Supplementary Notes

Supplementary Note 1. Pourbaix diagram calculation.

The Pourbaix diagram of RuO_2/CoO_x was constructed based on that of CoO_x (Supplementary Fig. 1). Specifically, RuO_2/CoO_x could form/dissolve through the following corrosion processes:

$$CoO_x{*}\ RuO_2 + 2H_2O \rightarrow CoO_x + RuO_4^{2-} + 4H^+ + 2e^- \quad (1)$$

or

$$CoO_x{*}\ RuO_2 + 2H_2O \rightarrow CoO_x + RuO_4^{-} + 4H^+ + 3e^- \quad (2)$$

with the free energy of the dissolution of $CoO_x{*}\ RuO_2$ as,

$$\Delta G_{CoO_x{*}\ RuO_2 \rightarrow CoO_x + RuO_4^{2-}} = \mu(CoO_x) + \mu(RuO_4^{2-}) + 4\mu(H^+) + 2\mu(e^-) - \mu(CoO_x{*}\ RuO_2) - 2\mu(H_2O) \quad (3)$$

or

$$\Delta G_{CoO_x{*}\ RuO_2 \rightarrow CoO_x + RuO_4^{-}} = \mu(CoO_x) + \mu(RuO_4^{-}) + 4\mu(H^+) + 3\mu(e^-) - \mu(CoO_x{*}\ RuO_2) - 2\mu(H_2O) \quad (4)$$

where $\mu(CoO_x) = \mu^0(CoO_x)$, $\mu(CoO_x{*}\ RuO_2) = \mu^0(CoO_x{*}\ RuO_2)$, $\mu(H_2O) = \mu^0(H_2O)$, and $\mu(e^-) = -eU$. $\mu^0(CoO_x)$, $\mu^0(CoO_x{*}\ RuO_2)$, and $\mu^0(H_2O)$ were directly calculated from DFT, and their computational models were shown in Supplementary Fig. 2. $\mu(aq) = \mu^0(aq) + k_B T\ln(\alpha(aq))$. The values of $\mu(RuO_4^{2-})$ and $\mu(RuO_4^{-})$ were referenced to the literatures[1,2]. For the proton (H^+), $H^+ + e^- \leftrightarrow 0.5\ H_2(g)$ has a standard free energy change of zero at the reduction potential U^0 = 0 V versus standard hydrogen electrode (SHE) under standard condition ($\alpha(H^+)$ = 1.0 M, pH = 0). Then $0.5\mu^0(H_2) = \mu^0(H^+) - eU^0(H^+/H_2)$. For $\mu(H^+)$ at any pH, $\mu(H^+) = 0.5\mu^0(H_2) - k_B T\ln 10\mathrm{pH}$[3].

Based on equations (3) and (4), the free energy of $CoO_x{*}\ RuO_2$ dissolution can be rewritten as

$$\Delta G_{CoO_x{*}\ RuO_2 \rightarrow CoO_x + RuO_4^{2-}} = \mu^0(CoO_x) + \mu^0(RuO_4^{2-}) + k_B T\ln\left(\alpha(RuO_4^{2-})\right) + 2\mu^0(H_2) - 4k_B T\ln 10\mathrm{pH} - 2eU_{SHE} - \mu^0(CoO_x{*}\ RuO_2) - 2\mu^0(H_2O) \quad (5)$$

or

$$\Delta G_{CoO_x{*}\ RuO_2 \rightarrow CoO_x + RuO_4^{-}} = \mu^0(CoO_x) + \mu^0(RuO_4^{-}) + k_B T\ln\left(\alpha(RuO_4^{-})\right) + 2\mu^0(H_2) - 4k_B T\ln 10\mathrm{pH} - 3eU_{SHE} - \mu^0(CoO_x{*}\ RuO_2) - 2\mu^0(H_2O) \quad (6)$$

When $\Delta G_{CoO_x{*}\ RuO_2 \rightarrow CoO_x + RuO_4^{2-}}$ or $\Delta G_{CoO_x{*}\ RuO_2 \rightarrow CoO_x + RuO_4^{-}} > 0$, RuO_2 is considered as electrochemically stable on CoO_x substrate. In the present work, the concentrations of ions are set to a concentration of 1×10^{-6} M.

Supplementary Note 2. Estimation of the proportion of interfacial Ru atoms to total Ru atoms in RuO_2/CoO_x catalyst.

As shown in Supplementary Fig. 15, the RuO_2 nanoparticles supported on CoO_x surface are cube-shaped with an average side length (a) of 2 nm. Note that the nearest neighboring Ru-Ru distance ($r_{\text{Ru-Ru}}$) measured in supplementary Fig. 15 is 3.14 Å. Therefore, the proportion of interfacial Ru atoms to total Ru atoms in RuO_2/CoO_x catalyst can be estimated to be ~15% according to the following equation:

$$N_{\text{interfacial Ru}}\% = \frac{N_{\text{interface}}}{N_{\text{total}}} = \frac{(a/r_{\text{Ru-Ru}})^2}{(a/r_{\text{Ru-Ru}})^3} \times 100\% \quad (7)$$

Supplementary Note 3. Calibration of turnover frequency (TOF) of RuO_2/CoO_x.

It was assumed that all the Ru ions in the hybrid catalyst are active sites in the OER process. The TOF of RuO_2/CoO_x was calculated as:

$$\mathrm{TOF} = \frac{\mathrm{O_2\ turnovers}}{\mathrm{Total\ Ru\ sites}} = \frac{N_{\mathrm{O_2}}}{N_{\mathrm{Ru}}} \quad (8)$$

where $N_{\mathrm{O_2}}$ and N_{Ru} are the O_2 turnover and the total Ru sites on the geometric area of the electrode, respectively. $N_{\mathrm{O_2}}$ can be calculated from the following equation:

$$N_{\mathrm{O_2}} = (J\,\frac{\mathrm{mA}}{\mathrm{cm^2}}) \times (\frac{\mathrm{1C/S}}{\mathrm{1000\ mA}}) \times (\frac{\mathrm{1\ mol\ e^-}}{\mathrm{96485\ C}}) \times (\frac{\mathrm{1\ mol\ O_2}}{\mathrm{4\ mol\ e^-}}) \times 6.02 \times 10^{23} \quad (9)$$

and N_{Ru} can be estimated by,

$$N_{\mathrm{Ru}} = \frac{m_{\mathrm{loading}} \times \mathrm{RuO_2\ wt\%} \times 6.023\times10^{23}}{M_{\mathrm{RuO_2}}} \quad (10)$$

where m_{loading} (0.25 mg cm^{-2}) is the loading mass of RuO_2/CoO_x on per geometrical area of the electrode, $M_{\mathrm{RuO_2}}$ is the molar mass of RuO_2. At an overpotential of 400 mV, the calculated TOF of RuO_2/CoO_x with 3.73 wt% RuO_2 is 3.61 s^{-1}. Note that the true TOF of the most active Ru/Co dual-atom sites (Supplementary Fig. 39) should be even higher because the number of Ru/Co dual-atom sites is much smaller than the total number of Ru sites on RuO_2/CoO_x.

Supplementary Note 4. OER RDS of RuO_2/CoO_x.

Notably, the generally accepted reaction steps[4] for OER under neutral conditions are

$$H_2O^* \rightarrow HO^* + H^+ + e^- \tag{11}$$

$$HO^* \rightarrow O^* + H^+ + e^- \tag{12}$$

$$O^* + H_2O\ (l) \rightarrow HOO^* + H^+ + e^- \tag{13}$$

$$HOO^* \rightarrow O_2 + H^+ + e^- \tag{14}$$

where * is the active site. As discussed the main text, for RuO_2/CoO_x, O–O bond formation (equation 13) is not the RDS. Our further calculation (Fig. 5c) reveals the RDS of RuO_2/CoO_x transfers to subsequent step of *OOH formation – that is, desorption of O_2 (equation 14).

Supplementary Note 5. Experimental validation of Ru/Co dual site as the active site for RuO_2/CoO_x.

First, we deposited RuO_2 nanoparticles on carbon black with the same particle size (~2 nm) as that on RuO_2/CoO_x (Supplementary Fig. 20c). As shown in Supplementary Fig. 37c, RuO_2/CoO_x exhibits a much higher current density than RuO_2 with the same RuO_2-mass loading, indicating the crucial role of interface in enhancing OER performance. Second, we prepared RuO_2/CoO_x with varied particle sizes (Supplementary Fig. 38) or loading masses of RuO_2. Assuming either surface Ru site or interfacial Ru-Co dual-atom site as the most active sites, the corresponding site number can be calculated. Specifically, the number of interfacial Ru/Co dual-atom sites ($N_{\text{interfacial Ru/Co dual-atom site}}$) can be numerically calculated by

$$N_{\text{interfacial Ru/Co dual-atom site}}=N_{\text{RuO}_2}\times n_{\text{interfacial Ru/Co dual-atom site}} \quad (15)$$

where N_{RuO_2} is the total number of RuO_2 nanoparticles deposited on CoO_x, and $n_{\text{interfacial Ru/Co dual-atom site}}$ is the number of interfacial Ru/Co dual-atom sites on each RuO_2 particle. Assuming that the RuO_2 nanoparticles are cube-shaped with an average side length (a) of 2 nm, N_{RuO_2} can be calculated by

$$N_{\text{RuO}_2}=\frac{m_{\text{RuO}_2}}{a^3\times\rho} \quad (16)$$

where m_{RuO_2} is the loaded RuO_2-mass and ρ is the density of RuO_2. Moreover, $n_{\text{interfacial Ru/Co dual-atom site}}$ can be obtained by

$$n_{\text{interfacial Ru/Co dual-atom site}}= 4\times(a/r_{\text{Ru-Ru}}) \quad (17)$$

where $r_{\text{Ru-Ru}}$ is the nearest neighboring Ru-Ru distance measured in supplementary Fig. 15. The number of surface Ru sites ($N_{\text{surface Ru site}}$) can be numerically calculated by

$$N_{\text{surface Ru site}}=N_{\text{RuO}_2}\times n_{\text{surface Ru site}} \quad (18)$$

where $n_{\text{surface Ru site}}$ is the number of surface of Ru sites on each RuO_2 particle, which can be calculated by

$$n_{\text{interfacial Ru/Co dual-atom site}}= 5\times(a/r_{\text{Ru-Ru}})^2 \quad (19)$$

We correlated $N_{\text{interfacial Ru/Co dual-atom site}}$ and $N_{\text{surface Ru site}}$ with the OER current density of these samples at 1.50 V_{RHE} (Supplementary Fig. 39). An adequate linear relationship was observed between $N_{\text{Ru/Co dual-atom sites}}$ and the OER current density (Supplementary Fig. 39c). Therefore, these collective results reveal that the interfacial Ru/Co dual-atoms are the most active OER sites on RuO_2/CoO_x catalyst.

Supplementary Figures

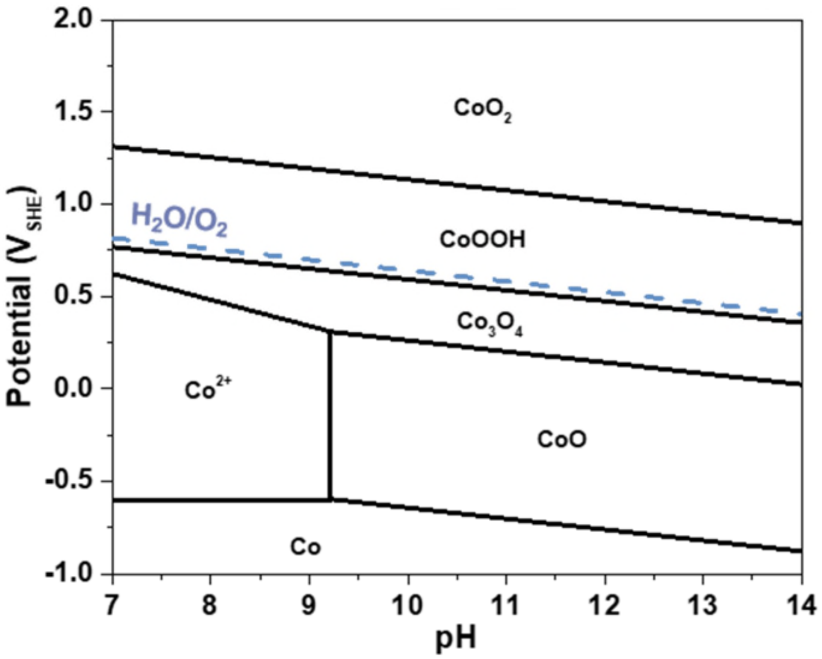

Supplementary Figure 1. Calculated Pourbaix diagram of CoO_x. The concentration of Co^{2+} is 10^{-6} M.

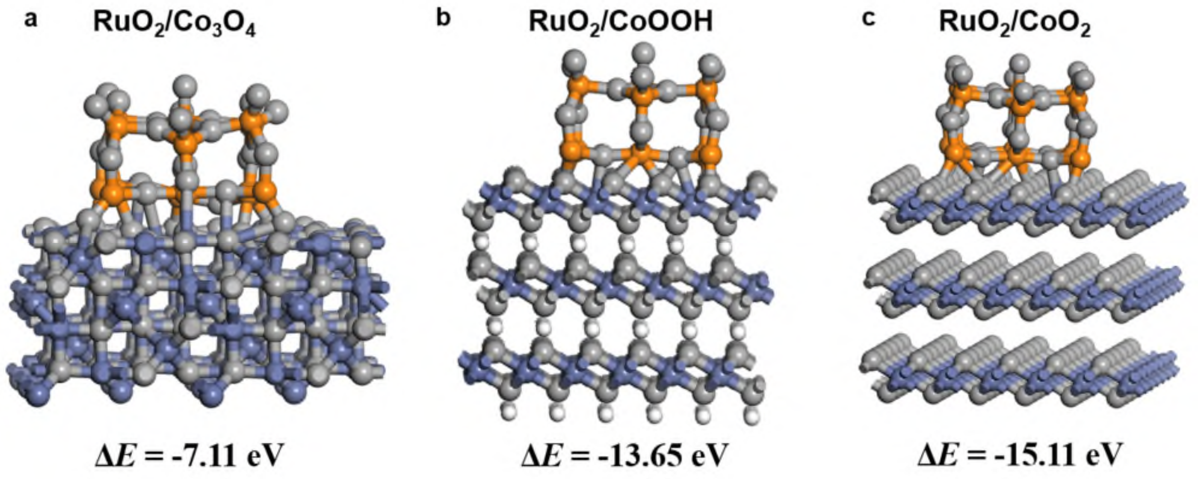

Supplementary Figure 2. Theoretical investigation on the adhesion energy of the RuO_2 cluster on CoO_x substrates. (a) RuO_2/Co_3O_4. (b) RuO_2/CoOOH. (c) RuO_2/CoO_2. Note that the adhesion energy (ΔE) was calculated by

$$\Delta E = E_{\mathrm{RuO_2/CoO}_x} - E_{\mathrm{RuO_2}} - E_{\mathrm{CoO}_x} \qquad (20)$$

where $E_{\mathrm{RuO_2/CoO}_x}$, $E_{\mathrm{RuO_2}}$, and E_{CoO_x} are the calculated energies for RuO_2/CoO_x, RuO_2 and CoO_x, respectively. The negative values of ΔE indicate a strong adhesion of RuO_2 to the CoO_x support. This undoubtedly decreases the driving force for RuO_2 dissolution, thus stabilizes RuO_2 in the hybrid catalyst.

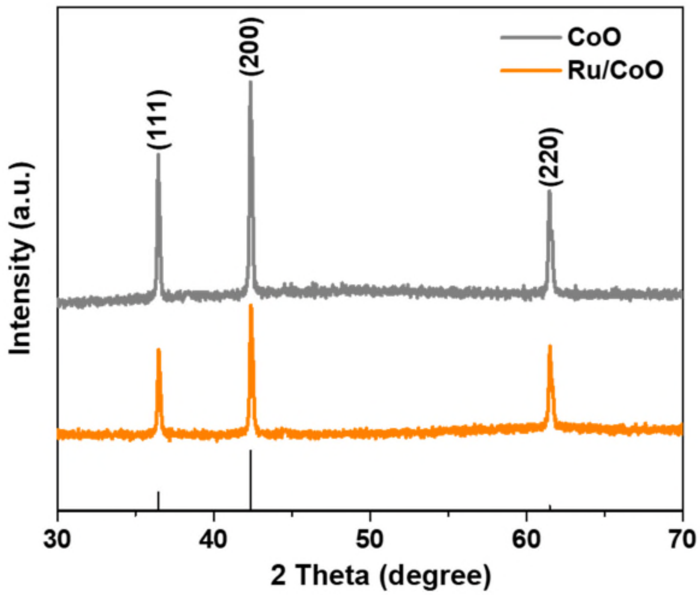

Supplementary Figure 3. X-ray diffraction (XRD) patterns of as-fabricated CoO and Ru/CoO.

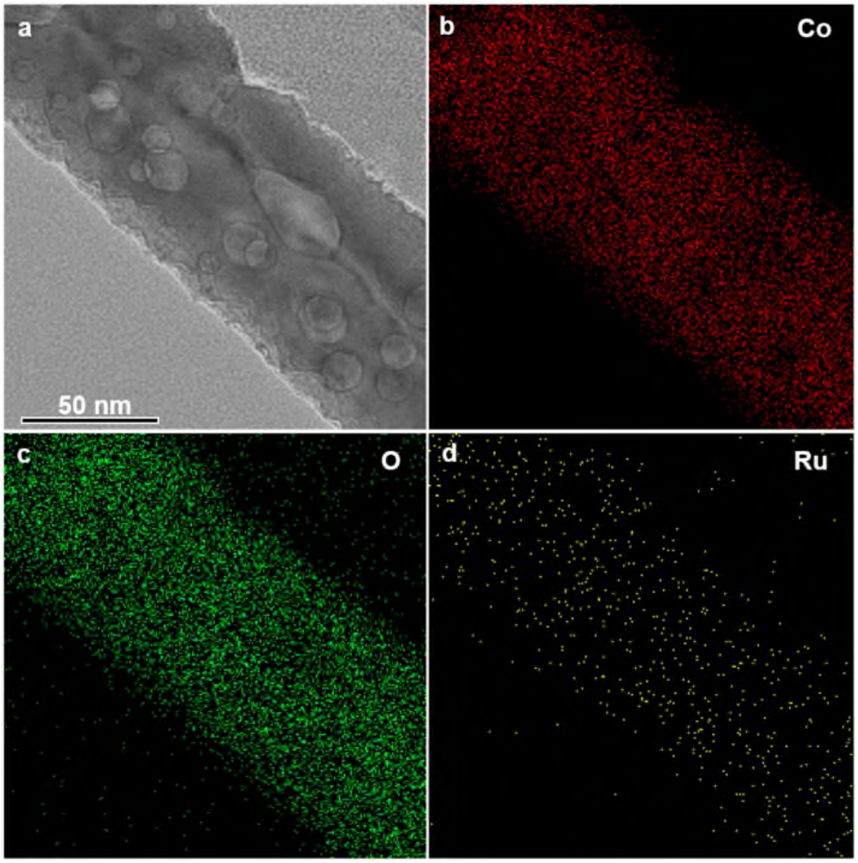

Supplementary Figure 4. Elemental analysis of the Ru/CoO. (a) Transmission electron microscopic (TEM) image. (b-d) EDS elemental mappings of Co, O, and Ru, respectively.

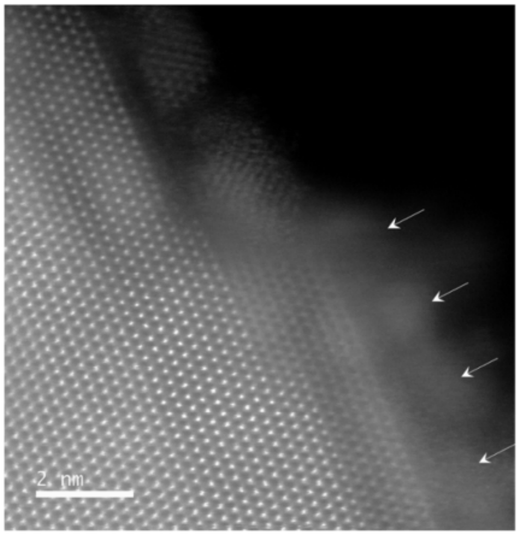

Supplementary Figure 5. HAADF-STEM image of Ru/CoO nanorod. Note that the different sharpness of the particles (shown by the white arrows) reflects their different positions relative to the focus of the electron beam. This reveals that the deposited Ru particles are distributed individually on CoO.

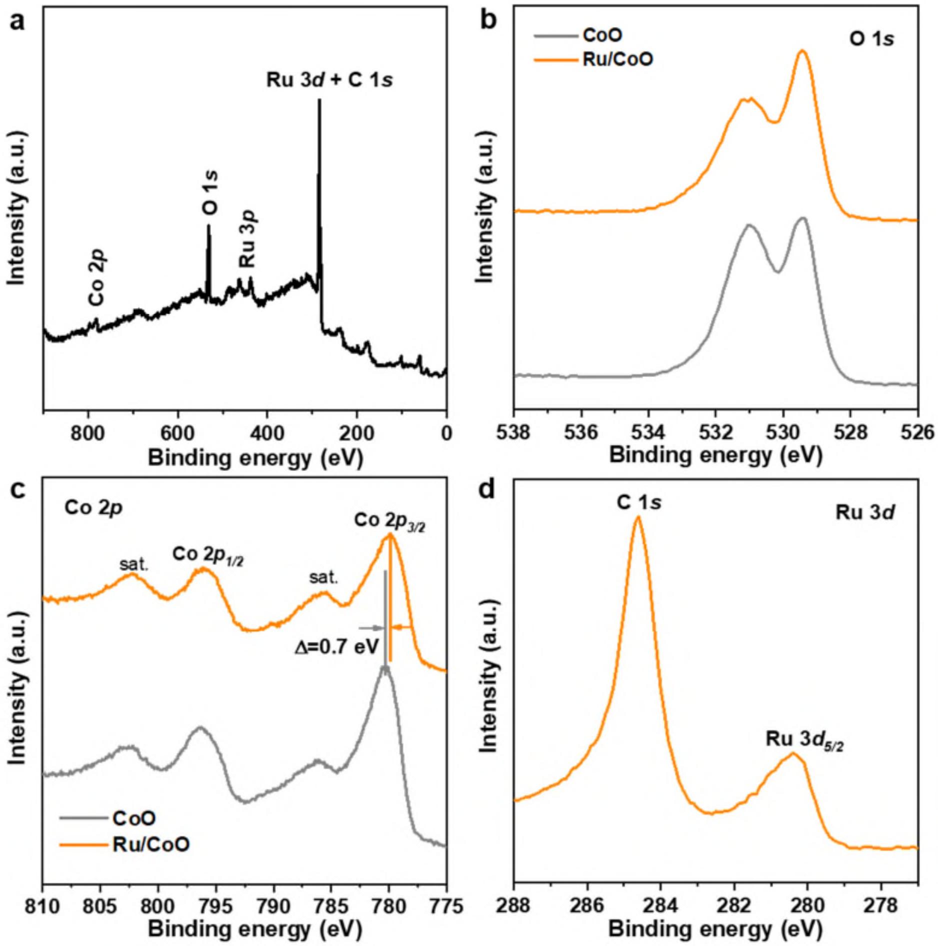

Supplementary Figure 6. X-ray photoelectron spectroscopic (XPS) characterizations of the Ru/CoO and CoO. (a) Survey spectrum. (b) O 1*s* spectra. (c) Co 2*p* spectra. (d) Ru 3*d* spectrum.

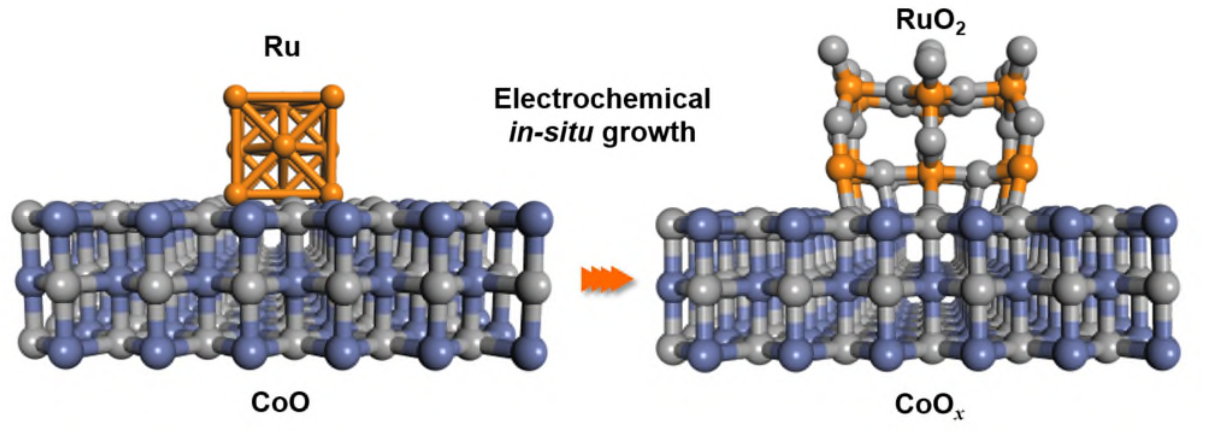

Supplementary Figure 7. Schematic diagram of the synthesis of RuO_2/CoO_x via an *in situ* electrochemical oxidation process.

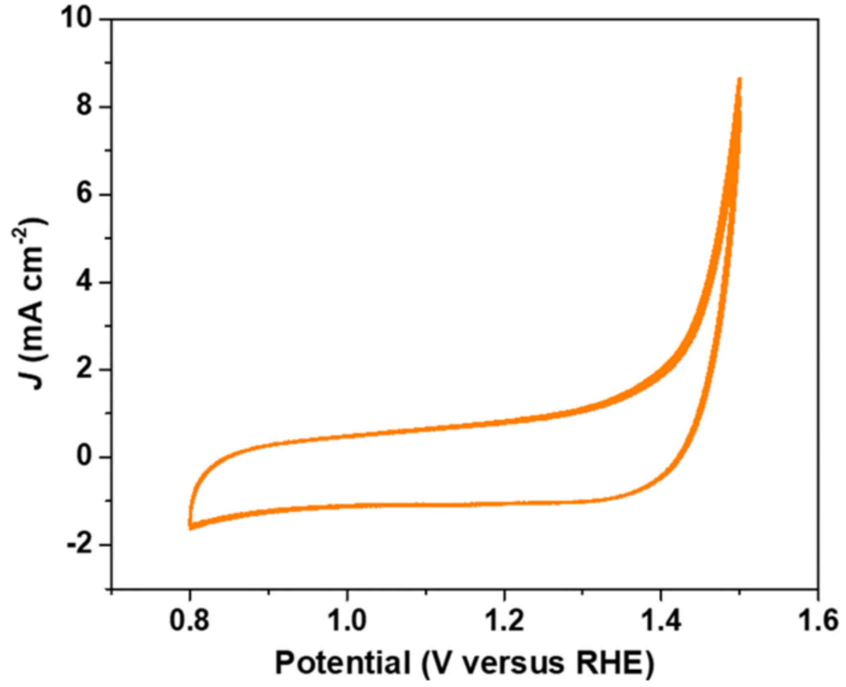

Supplementary Figure 8. Cyclic voltammetry (CV) curves of the electrochemical oxidization of Ru/CoO to form the RuO_2/CoO_x.

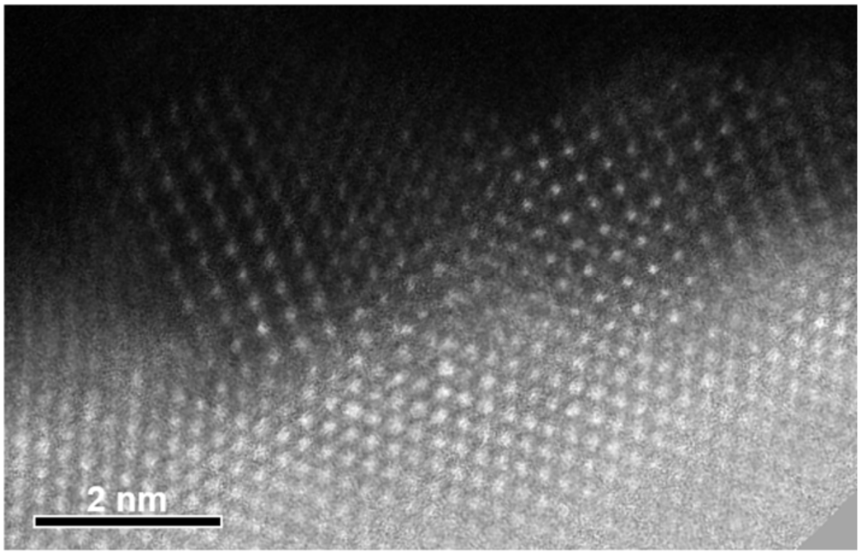

Supplementary Figure 9. High-angle annular dark-field scanning transmission electron microscopy (HAADF-STEM) image of the RuO_2/CoO_x.

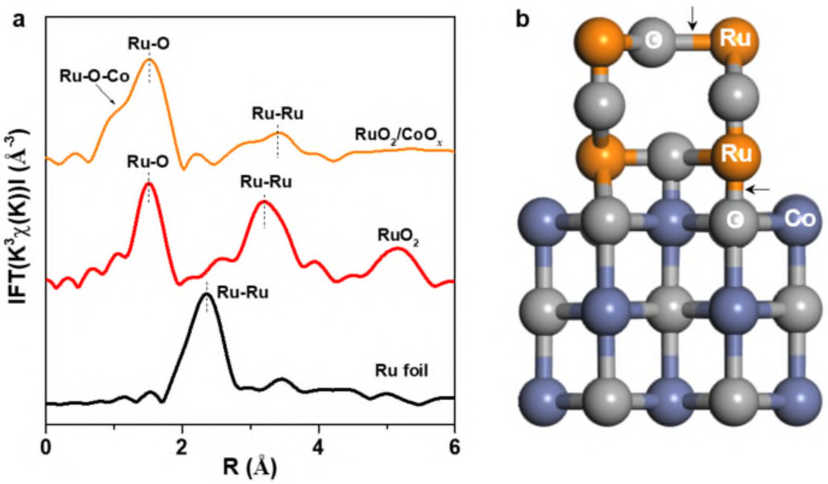

Supplementary Figure 10. (a) Fourier transform extended X-ray absorption fine structure (FT-EXAFS) spectra of RuO_2/CoO_x, RuO_2 and Ru foil. (b) Schematic diagram showing the Ru-O bond in bulk RuO_2 and Ru-O bond in interfacial Ru-O-Co. As shown in (a), a new peak assigned to Ru-O-Co bonds (with shorter Ru-O distance in Ru-O-Co than that in RuO_2) appears in the FT-EXAFS spectrum of RuO_2/CoO_x.

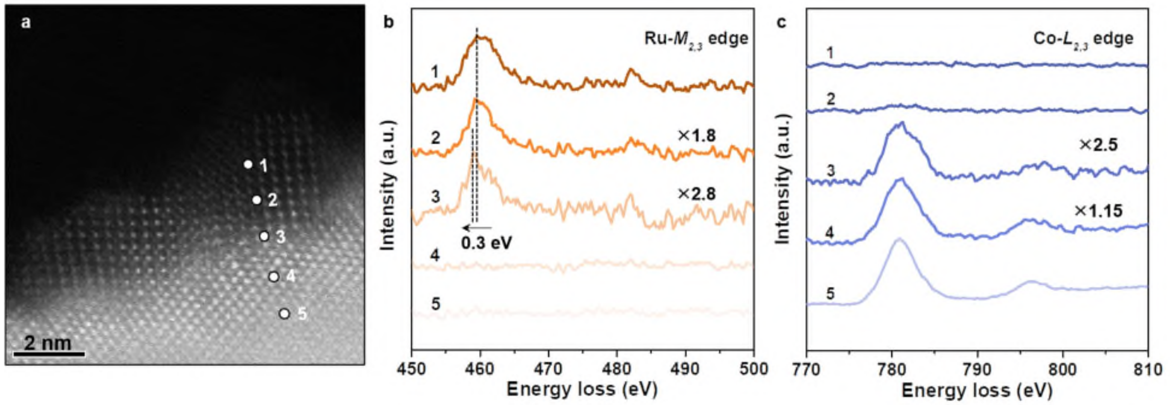

Supplementary Figure 11. Electron energy-loss spectroscopy (EELS) analysis of the RuO_2/CoO_x across the interface. (a) HAADF-STEM image. Panel b and c show the EELS spectra of Ru-$M_{2,3}$ and Co-$L_{2,3}$ edge across the interface from point 1 to point 5 in (a), respectively.

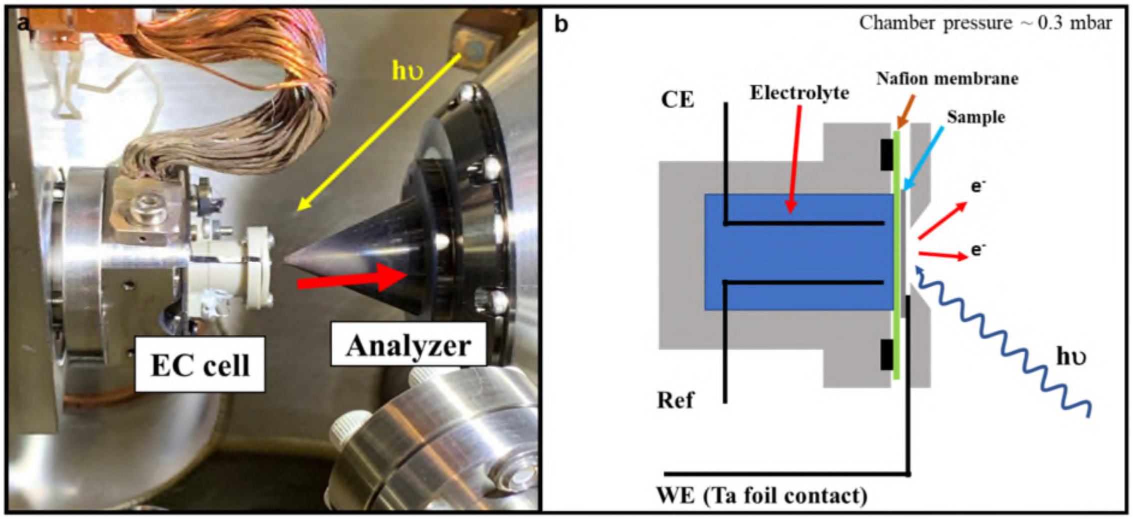

Supplementary Figure 12. Schematic diagram of the *in situ* XPS analysis of RuO_2/CoO_x. (a) Photograph of the electrochemical cell (EC) in the analysis chamber of ambient pressure XPS end station. The analysis chamber pressure is ~0.3 mbar. (b) Scheme of the EC cell.

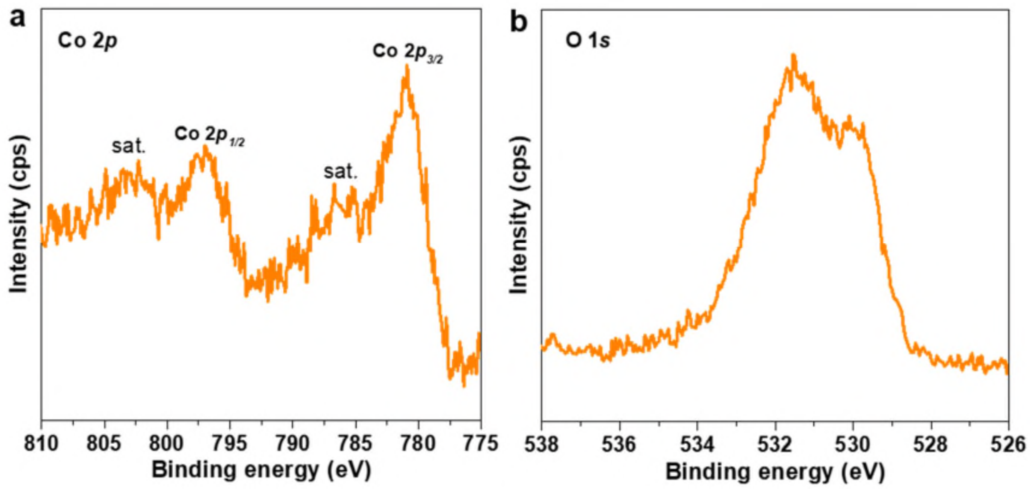

Supplementary Figure 13. (a) Co 2*p* and (b) O 1*s* XPS spectra of the CoO_x collected in the *in situ* measurements.

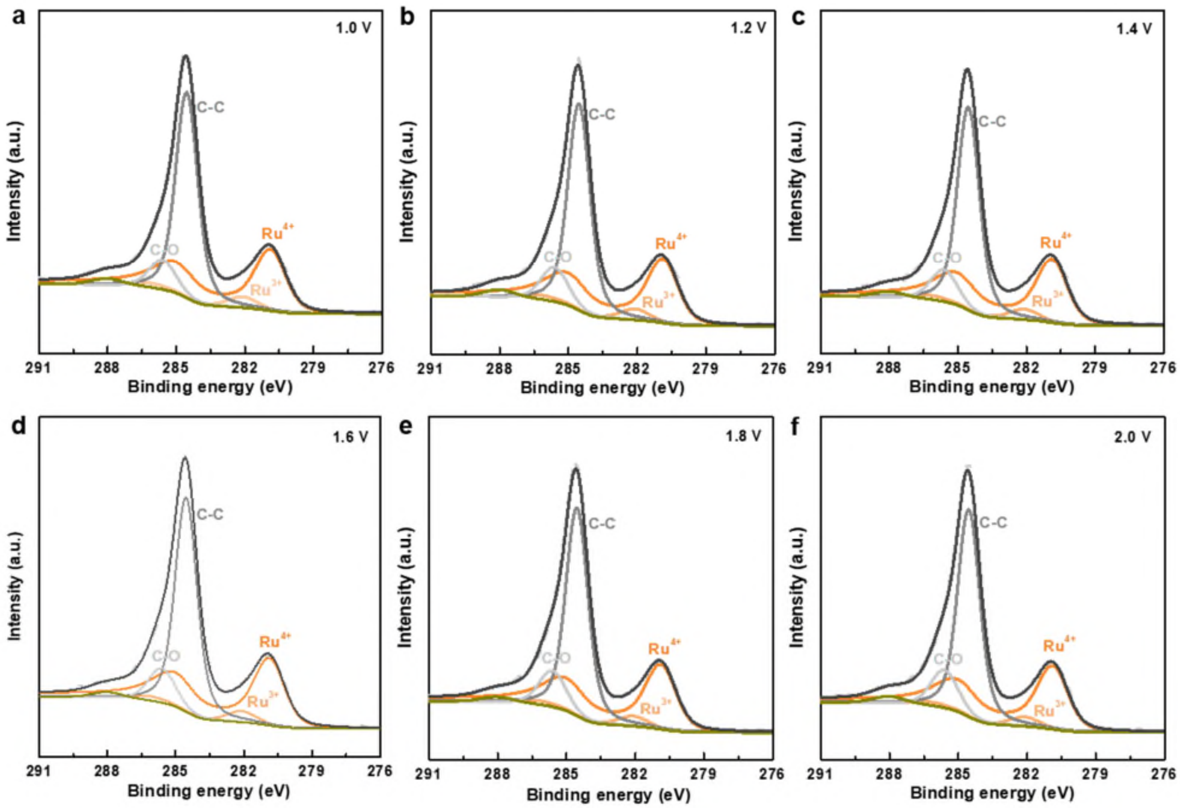

Supplementary Figure 14. *In situ* Ru 3*d* XPS spectra for RuO_2/CoO_x recorded at applied potential during 1.00-2.00 V_{RHE}. As shown, Ru^{4+} and Ru^{3+} peaks are centered at 280.9 eV and 282.1 eV[5].

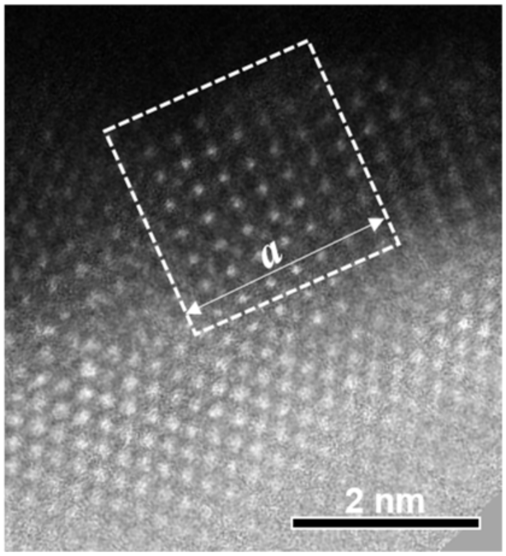

Supplementary Figure 15. HAADF-STEM image of the RuO_2/CoO_x. It shows that the RuO_2 nanoparticles supported on CoO_x surface are cube-shaped with an average side length (*a*) of 2 nm.

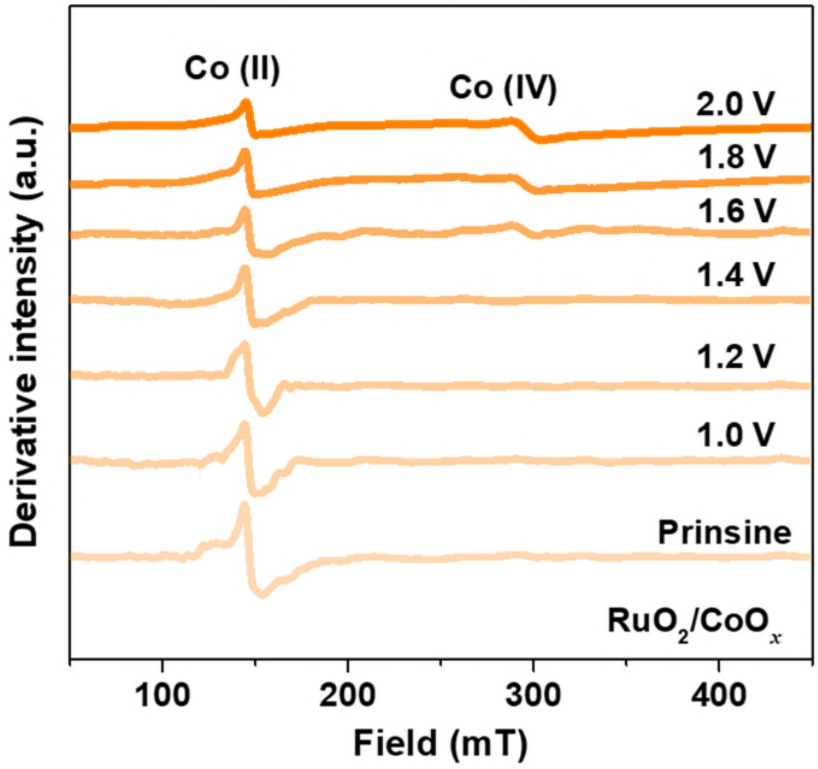

Supplementary Figure 16. EPR spectra of RuO_2/CoO_x at different potentials in 1.0 M phosphate buffered saline (PBS). Note that the potentials were referenced to RHE. Signals with g_{eff} of 4.22[6,7] and 2.15[8-11] correspond to Co^{2+} and Co^{4+}, respectively. As can be seen, the content of Co^{2+} decreases with increasing the applied potential. Co^{4+} species appear at E =1.60 V_{RHE}. The quantitative EPR results are provided in Fig. 3d.

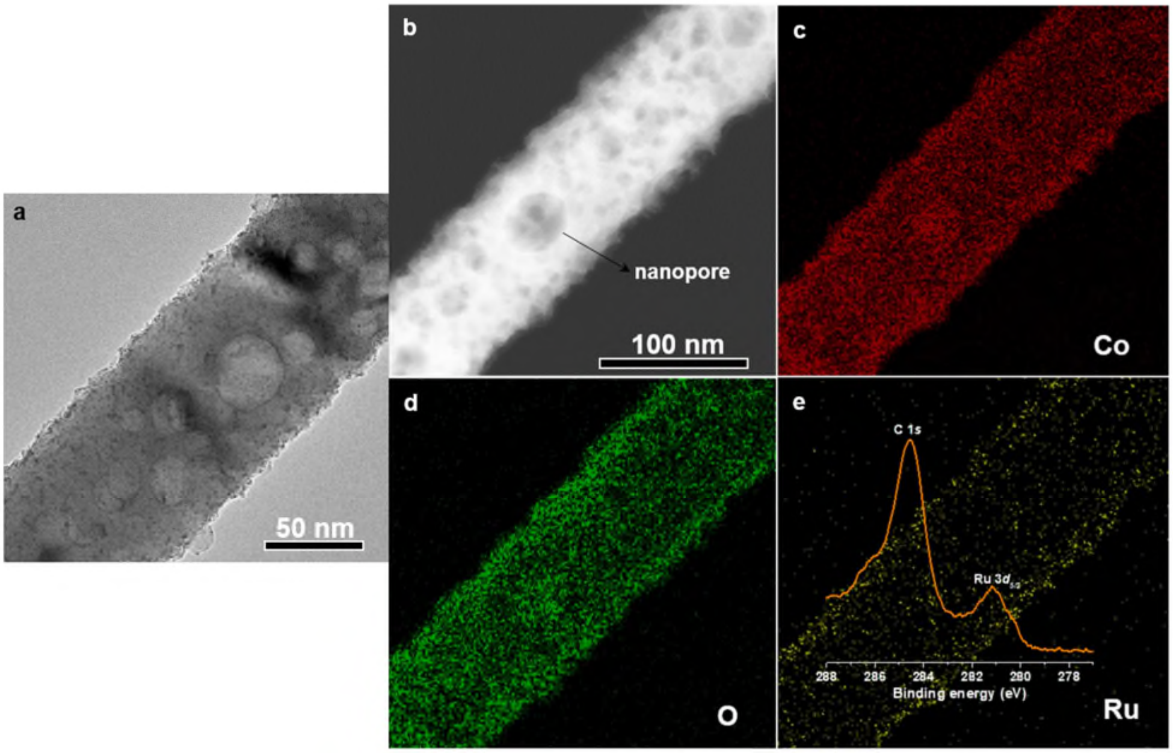

Supplementary Figure 17. Structural characterizations of the RuO_2/CoO_x after 20 h continuous test at 1.80 V_{RHE}. (a) TEM image. (b)-(e) HADDF-STEM image and corresponding elemental mappings of Co, O and Ru, respectively. Notably, the nanopores were generated by the release of lattice strain between the template ZnO and CoO during the cation exchange process[12]. The inset of (e) shows the Ru 3*d* XPS spectrum of RuO_2/CoO_x after stability test. As shown, the structural integrity of the RuO_2/CoO_x is well preserved and RuO_2 nanoparticles are still distributed on CoO_x after the OER process.

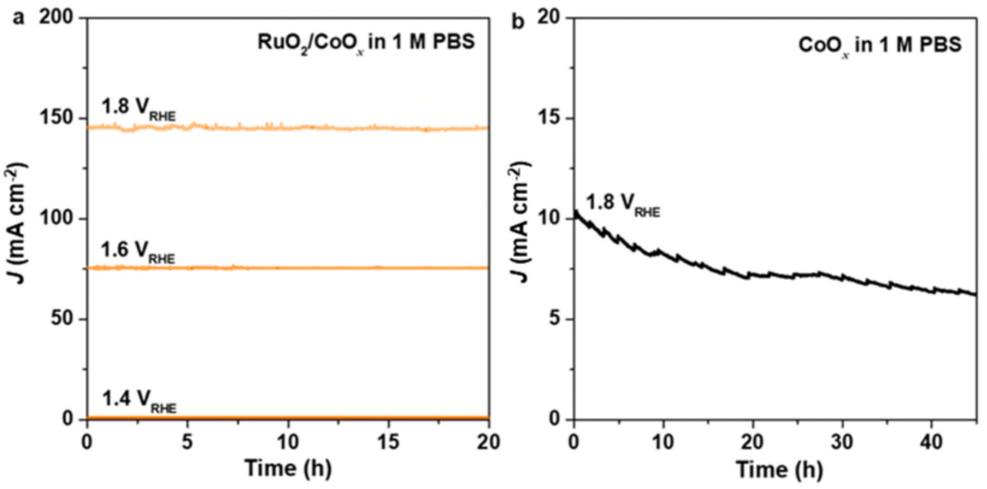

Supplementary Figure 18. Long-term stability tests of the RuO_2/CoO_x in neutral solution. (a) Current density (*I*)-time (*t*) curves of the RuO_2/CoO_x at varied potentials. (b) *I*-*t* curve of the reference CoO_x at 1.80 V_{RHE}. The much larger current density of the RuO_2/CoO_x (~150 mA cm^{-2}) than those of pristine CoO_x (~10 mA cm^{-2}) and RuO_2 (20~30 mA cm^{-2}, Supplementary Fig. 22a) at the same applied potential (1.80 V_{RHE}) confirms the enhanced activity of the RuO_2/CoO_x hybrid catalyst.

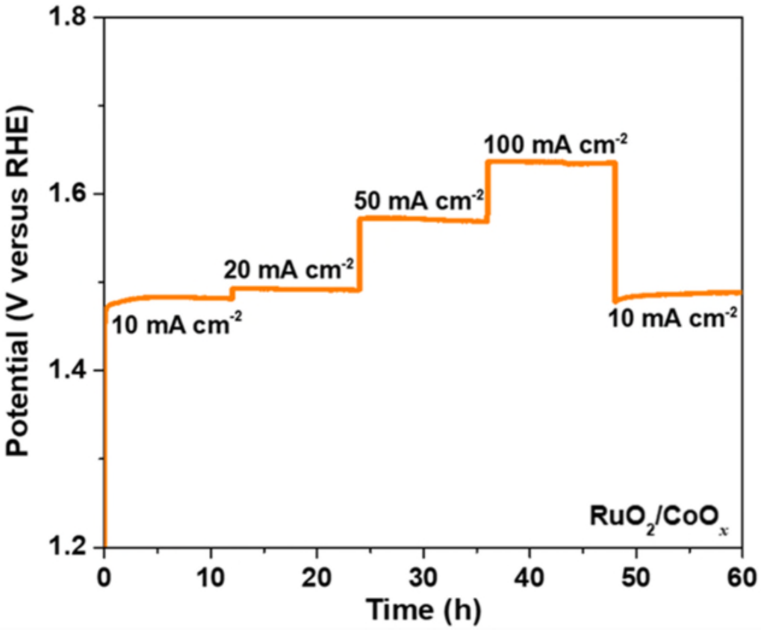

Supplementary Figure 19. Dynamic stability test of RuO_2/CoO_x with current densities from 10 to 100 mA cm^{-2} in 1.0 M PBS.

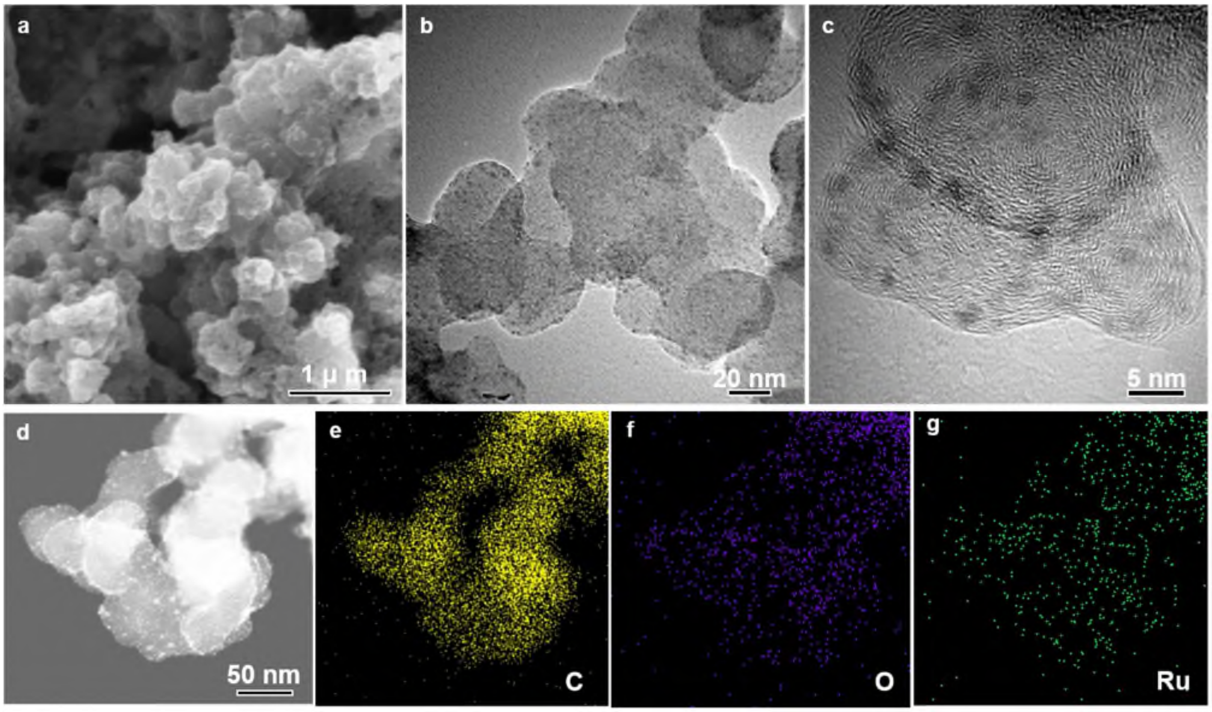

Supplementary Figure 20. Characterization of the pristine RuO_2 deposited on carbon black. (a) SEM image, (b) and (c) Low- and high-magnification TEM images, respectively. (d)-(g) HAADF-STEM image and corresponding elementral mappings of C, O and Ru, respectively.

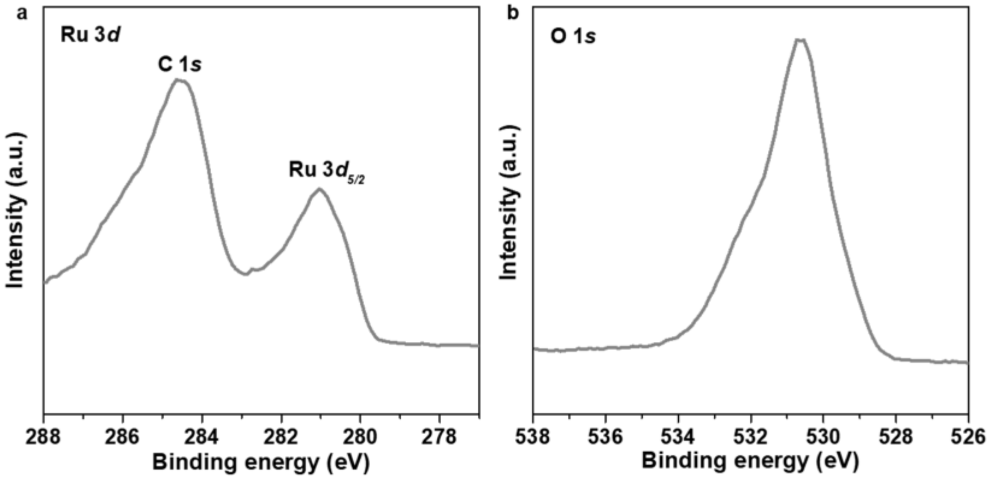

Supplementary Figure 21. (a) Ru 3*d* and (b) O 1*s* XPS spectra of the pristine RuO_2 deposited on carbon black.

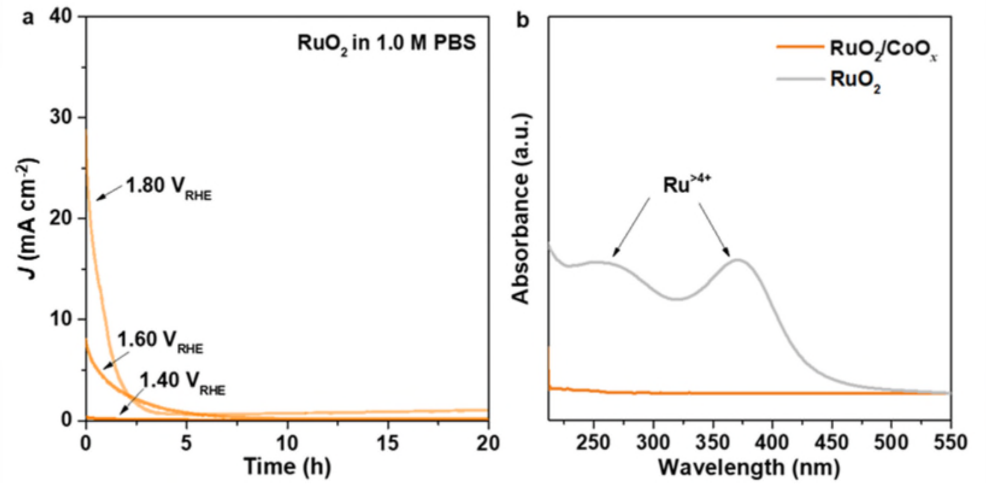

Supplementary Figure 22. Stability tests of RuO_2 in neutral solution. (a) *I*-*t* curves of RuO_2 at 1.40, 1.60 and 1.80 V_{RHE}, all showing significant current density attenuations during test. (b) UV-Vis spectra of the 1.0 M PBS electrolytes after the RuO_2 and RuO_2/CoO_x were tested for 20 h. As illustrated, the UV-Vis spectrum of RuO_2 electrolyte shows obvious peaks at 254 and 371 nm, corresponding to hydrated Ru^{n+} ions (n>4)[13,14], which demonstrates the dissolution of RuO_2 during OER. In contrast, no absorption peaks can be observed in the spectrum of RuO_2/CoO_x electrolyte.

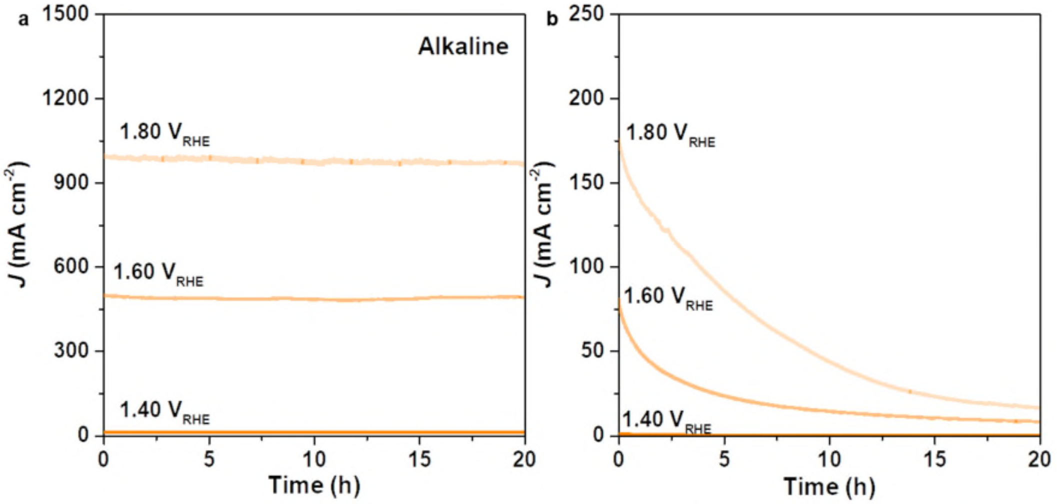

Supplementary Figure 23. Long-term stability tests of (a) RuO_2/CoO_x and (b) RuO_2 at varied potentials in alkaline solution. Note that catalysts with identical mass of 2.0 mg cm^{-2} were loaded on nickel form for stability test.

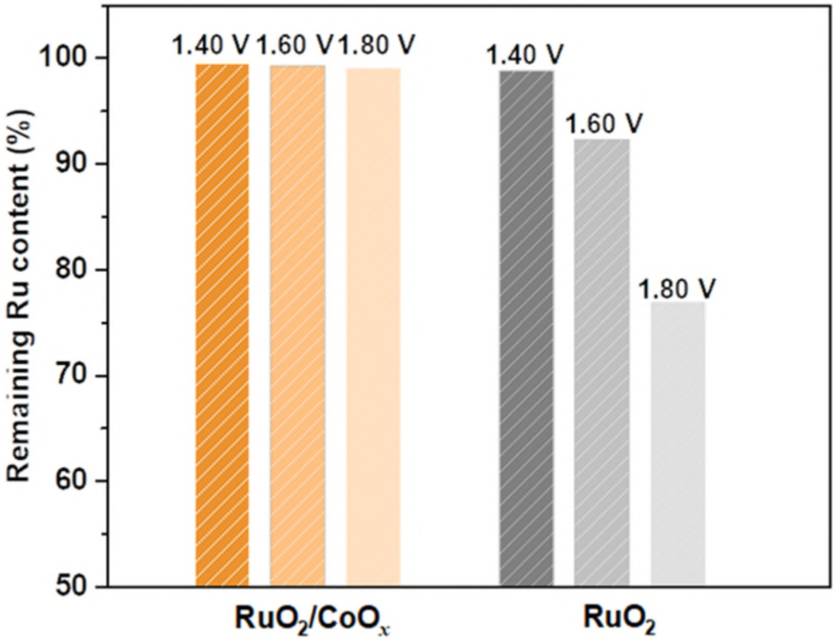

Supplementary Figure 24. Remaining Ru contents in the RuO_2/CoO_x and RuO_2 after continuously tested at different potentials for 20 h in alkaline electrolytes. Note that the potentials were referenced to RHE.

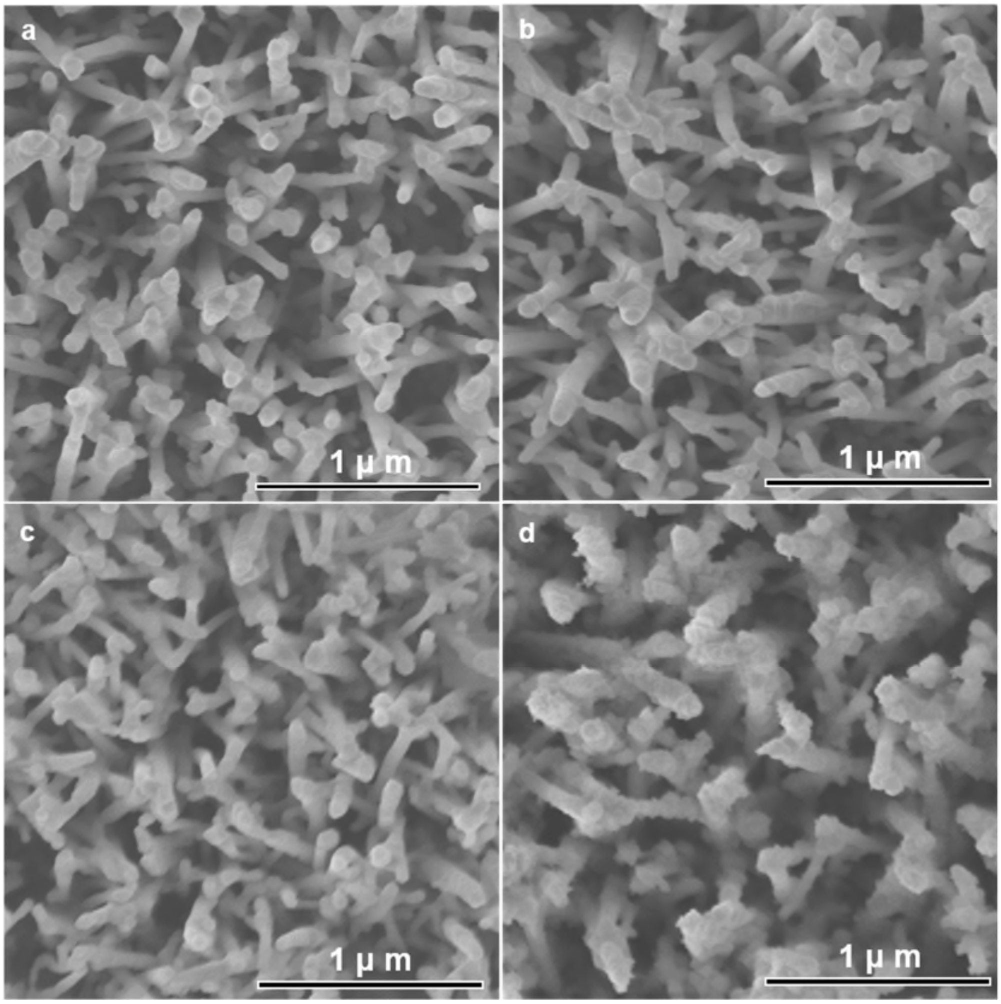

Supplementary Figure 25. SEM characterizations of RuO_2/CoO_x with different RuO_2 mass loadings on per cm^2 electrode. (a) 3 μg cm^{-2}. (b) 6 μg cm^{-2}. (c) 17 μg cm^{-2}. (d) 30 μg cm^{-2}.

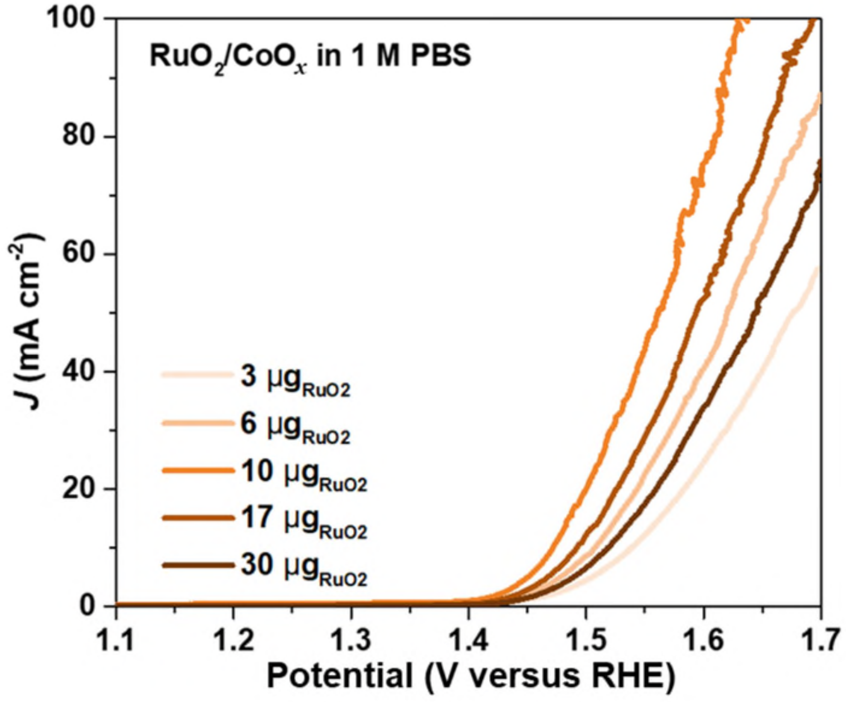

Supplementary Figure 26. Polarization curves of the RuO_2/CoO_x with different RuO_2-masses on per cm^2 electrode in neutral electrolyte. It shows that the RuO_2/CoO_x with RuO_2 loading of 10 μg exhibits the optimal OER activity.

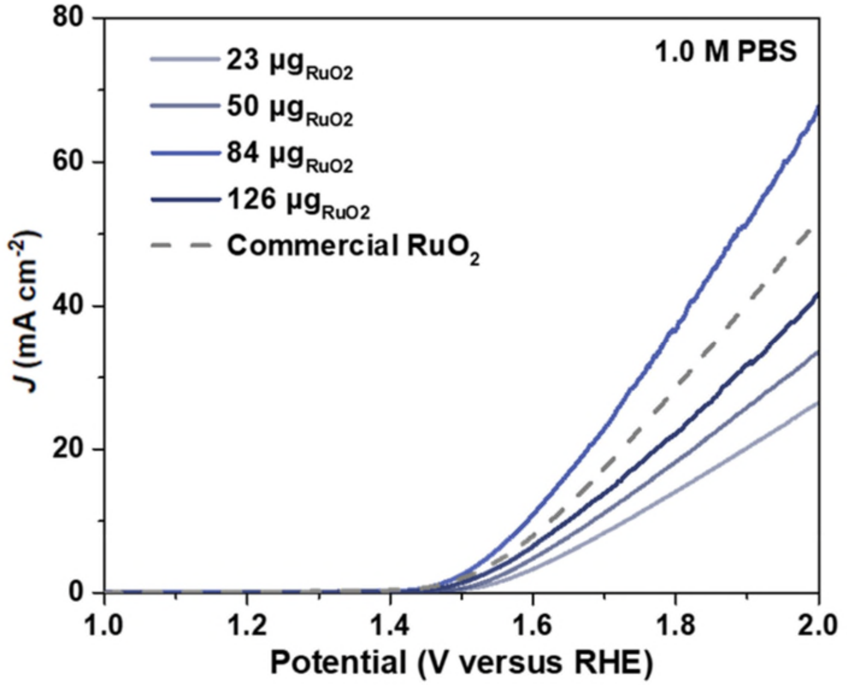

Supplementary Figure 27. Polarization curves of RuO_2 deposited on carbon black with different RuO_2-masses on per cm^2 electrode in neutral electrolyte with the commercial RuO_2 as reference. It shows that the catalyst with RuO_2 loading of 84 μg cm^{-2} exhibits the optimal OER activity, which is better than the commercial RuO_2 catalyst (0.255 mg cm^{-2}). Therefore, this RuO_2 sample was used as the control sample in this work.

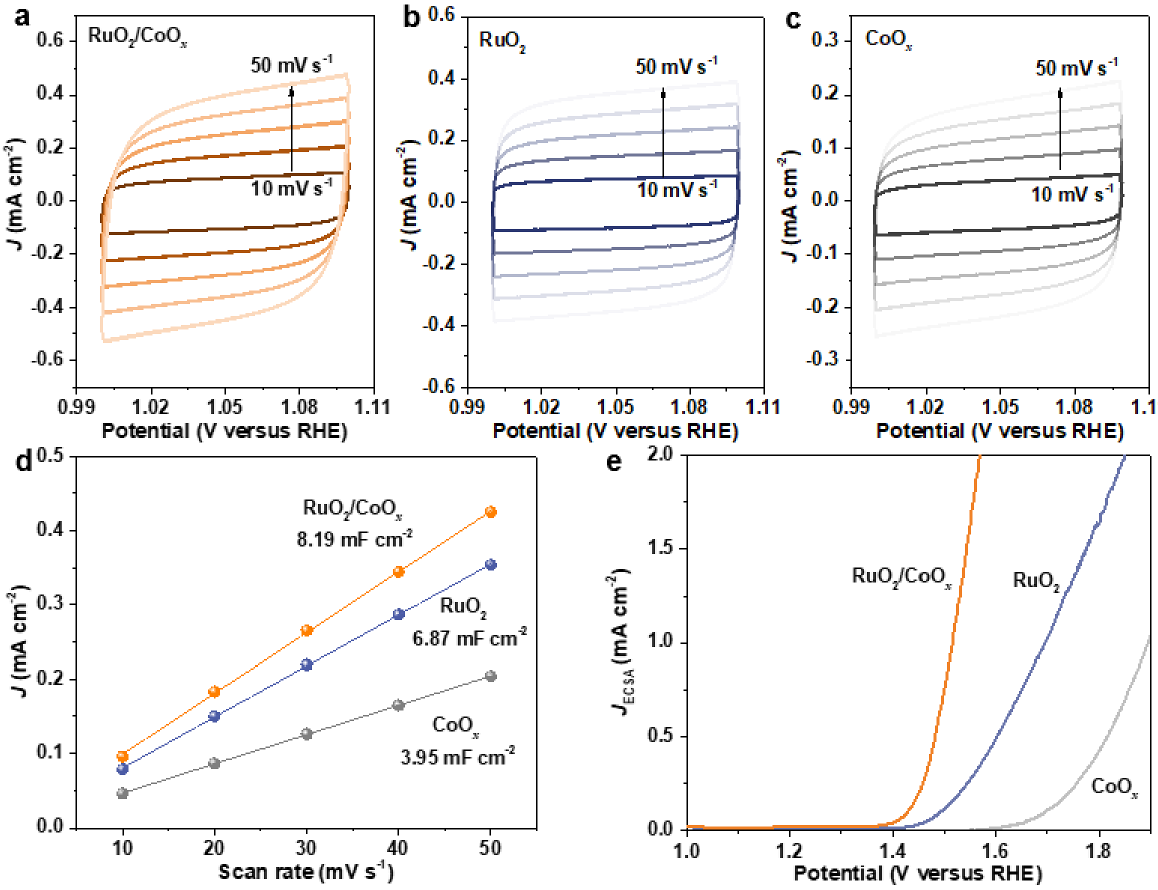

Supplementary Figure 28. (a-c) Plots of current density (J) versus scan rate for RuO_2/CoO_x, RuO_2 and CoO_x, respectively. (d) Determined double-layer capacitance (C_{dl}) for RuO_2/CoO_x, RuO_2 and CoO_x. Note that the electrochemically active surface area (ECSA) of the catalyst can be calculated by $\text{ECSA} = \frac{C_{dl}}{C_s} \times S_{geometric}$, where C_s is 60 μF·cm^{-2}, $S_{geometric}$ is the geometric area of the glassy-carbon electrode, and C_{dl} was the measured electrical double-layer capacitor of the catalyst. (e) Intrinsic activity of RuO_2/CoO_x, RuO_2 and CoO_x. Note that J_{ECSA} is obtained by normalizing the OER current density to the calculated ECSA.

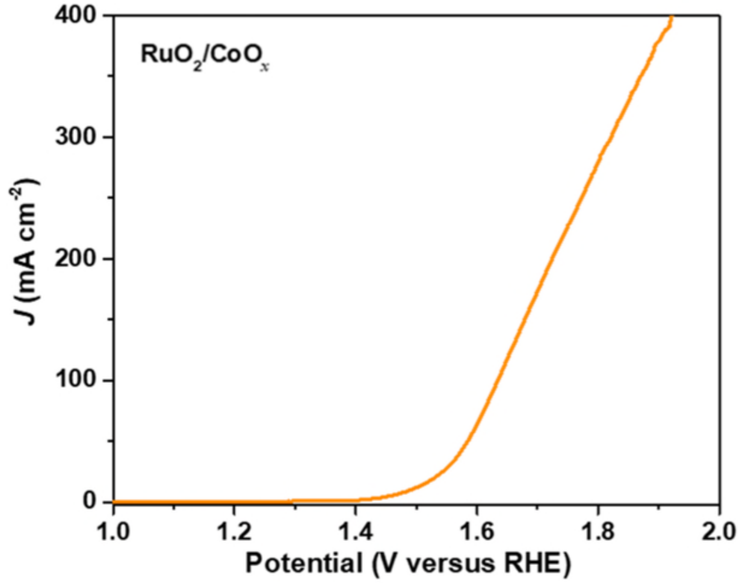

Supplementary Figure 29. OER polarization curve of RuO_2/CoO_x with a catalyst loading of 1.5 mg cm^{-2} on nickel form in neutral electrolyte (1.0 M PBS).

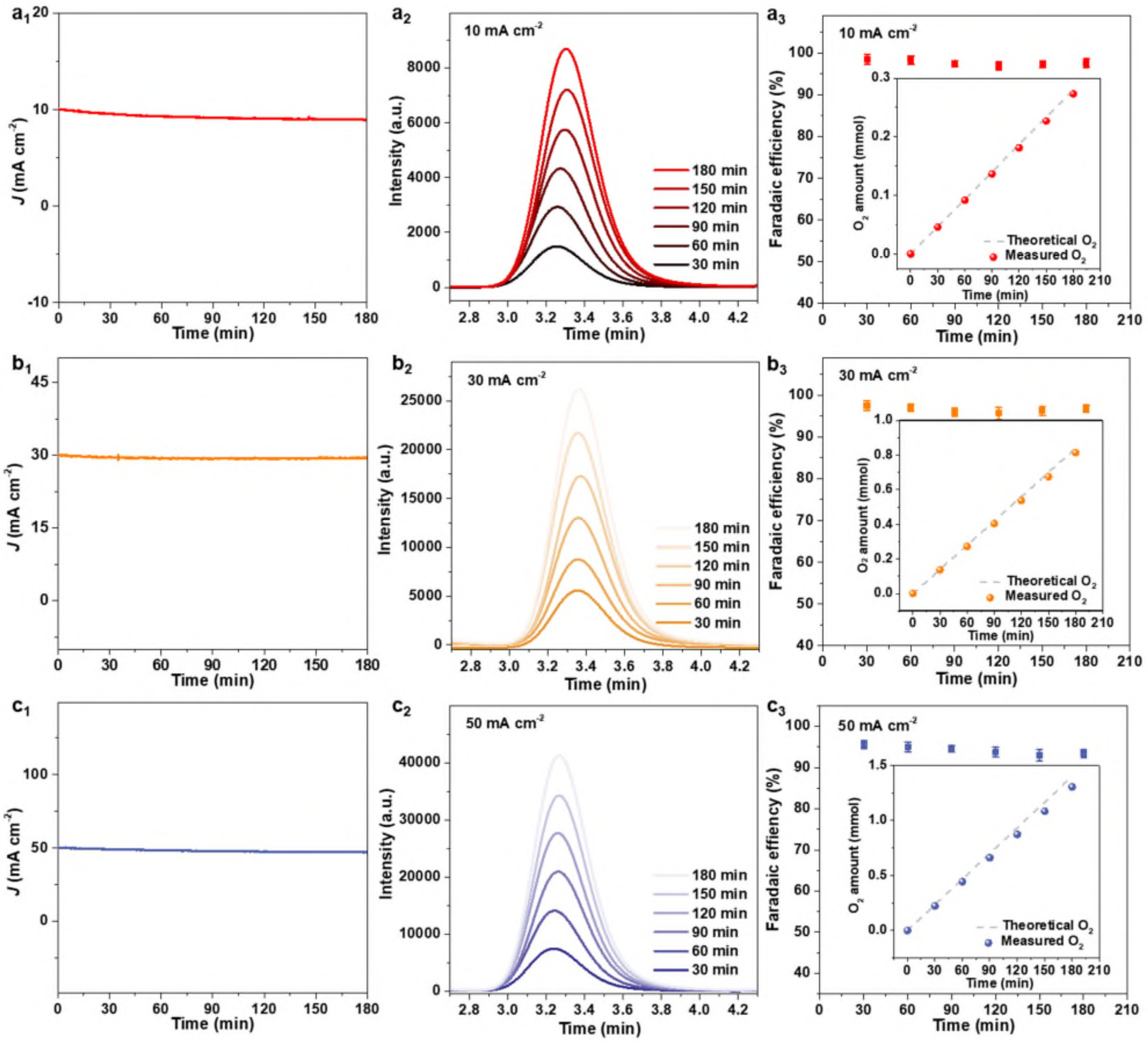

Supplementary Figure 30. Faradaic efficiency measurement of RuO_2/CoO_x at 10, 30, and 50 mA cm^{-2}. (a_1), (b_1), and (c_1) chronoamperometric curves obtained for gas chromatography (GC) tests. (a_2), (b_2), and (c_2) O_2 GC signals. (a_3), (b_3), and (c_3) Faradaic efficiencies for O_2 generation on RuO_2/CoO_x, with the insets showing the amount of experimentally collected O_2 and theoretically calculated O_2 based on Faraday's law[15],

$$\text{FE}(O_2) = n_{\text{O2-measured}}/n_{\text{O2-calculated}} = 4F\times n_{\text{O2-measured}}/Q \quad (21)$$

where $n_{\text{O2-measured}}$ and $n_{\text{O2-calculated}}$ are the experimentally measured and theoretically calculated O_2 amount, respectively, F is the Faraday constant (96500 C mol^{-1}), and Q is the total charge, which was calculated by integrating the chronoamperometric curve. These results show that RuO_2/CoO_x is highly selective for OER in neutral environments.

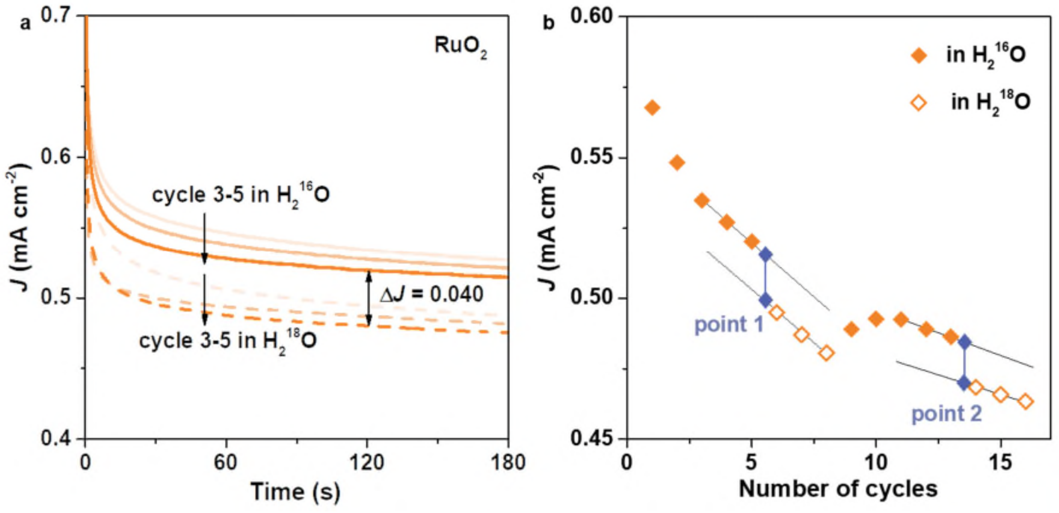

Supplementary Figure 31. Kinetic isotope effect (KIE) of RuO_2 in neutral electrolyte. (a) Current density (*J*)-time curves with multiple cycles. (b) Average current density ($J_{average}$) during the last 2 minutes for each cycle. The KIE value was estimated from the ratio of the data of the blue points in the two fitted line in $H_2{}^{16}O$ and $H_2{}^{18}O$.

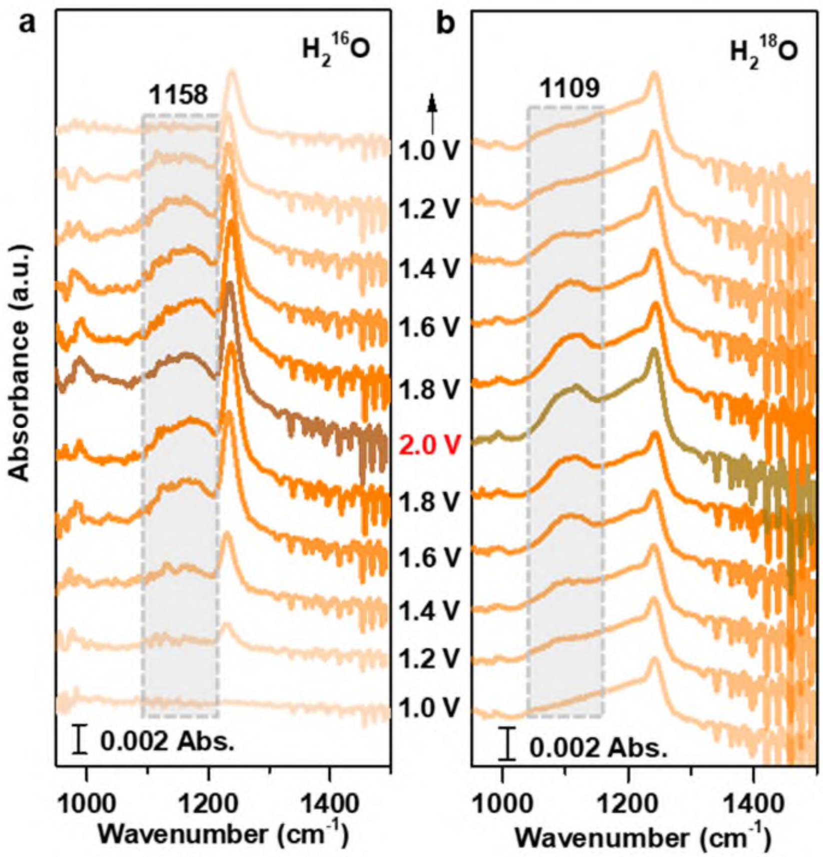

Supplementary Figure 32. *In situ* surface-enhanced IR spectra of RuO_2/CoO_x in neutral electrolyte prepared by (a) $H_2{}^{16}O$ and (b) $H_2{}^{18}O$.

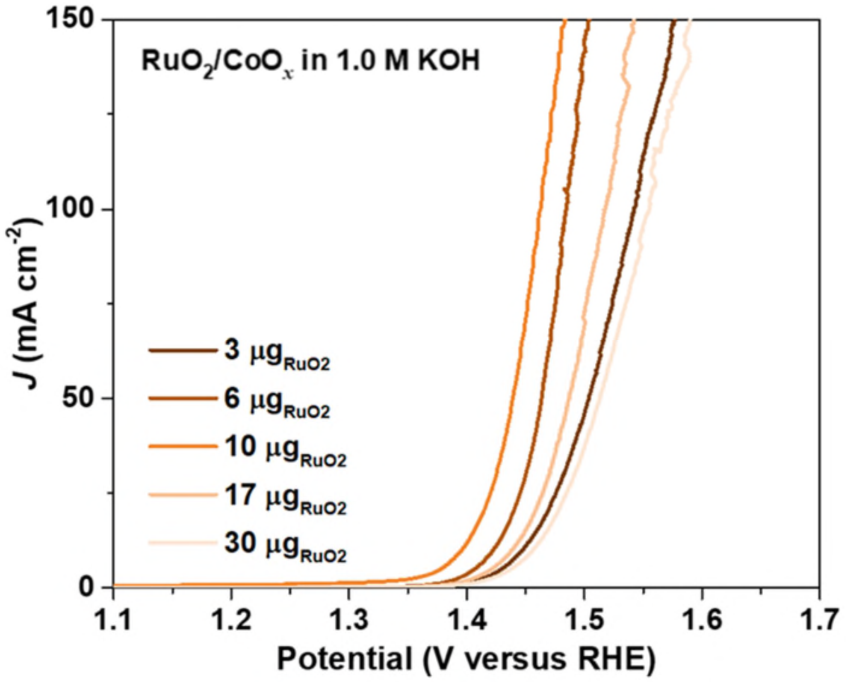

Supplementary Figure 33. Polarization curves of the RuO_2/CoO_x with different RuO_2-masses on per cm^{-2} electrode in alkaline electrolyte. It shows that the RuO_2/CoO_x with RuO_2 loading of 10 μg exhibits the optimal OER activity.

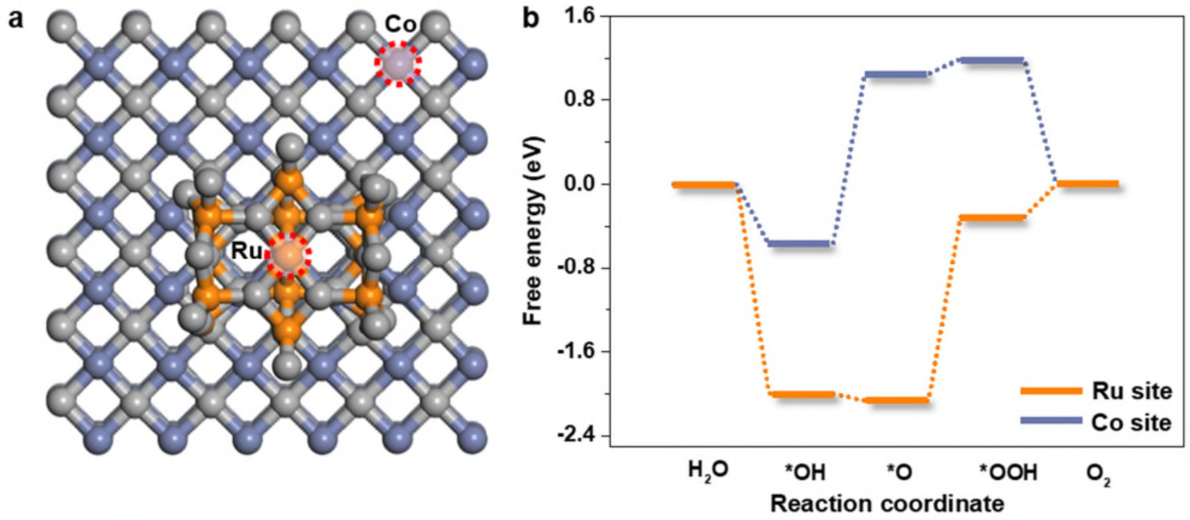

Supplementary Figure 34. Theoretical investigations on the possible active sites of the RuO_2/CoO_x. (a) Selected Ru and Co sites that are away from the interface. (b) Corresponding calculated OER free energy diagrams of the selected Ru and Co sites in (a), confirming that the sites away from the interface are less active than the interfacial Ru/Co dual-atom sites.

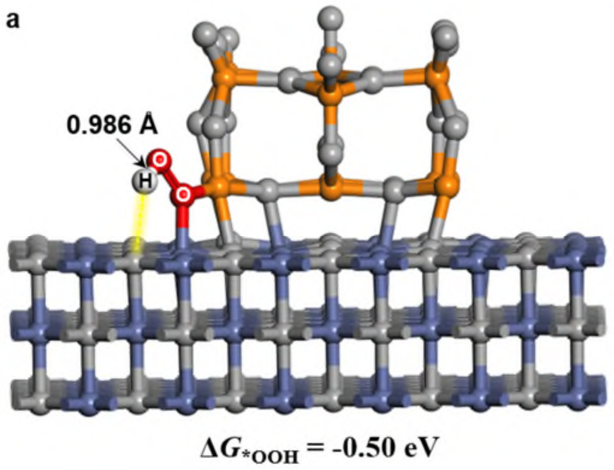

Supplementary Figure 35. Theoretical investigation of *OOH formation on (a) RuO_2/CoO_x and (b) RuO_2. As seen, the unique adsorption configuration of *OO–H···O facilitates the stabilization of *OOH at the interfacial Ru/Co dual-atom site with a more favarable ΔG_{*OOH} of -0.50 eV.

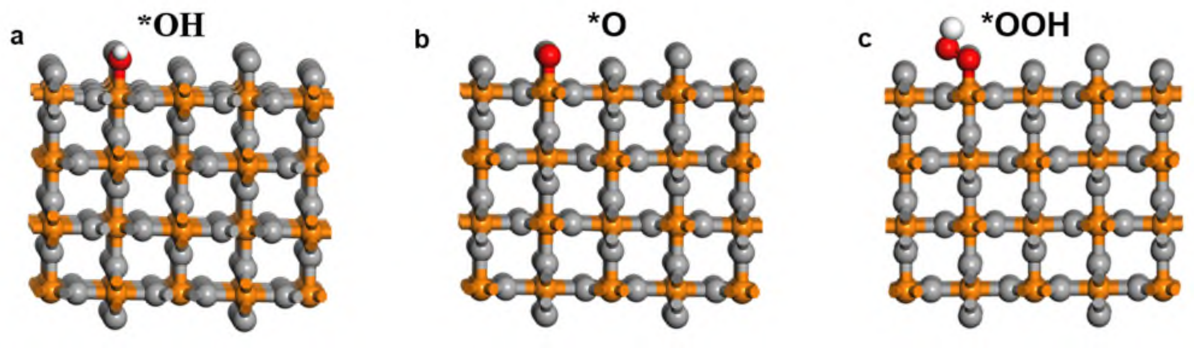

Supplementary Figure 36. Computationally-optimized geometric structures of oxygen intermediates adsorbed on the RuO_2 surface. (a) *OH. (b) *O. (c) *OOH.

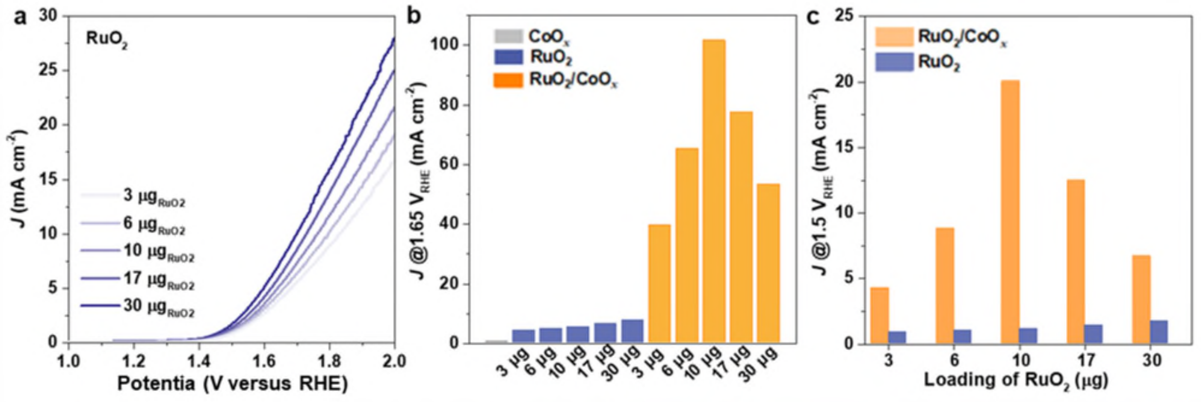

Supplementary Figure 37. (a) Polarization curves of the RuO_2 deposited on carbon black with different RuO_2-masses on per cm^2 electrode in neutral electrolyte. (b) Comparison of current density (J) of CoO_x, RuO_2/CoO_x and RuO_2 at 1.65 V_{RHE}. The data for RuO_2/CoO_x and RuO_2 with different RuO_2-masses are presented. Note that the current densities of RuO_2/CoO_x are significantly higher than those of RuO_2 and CoO_x, indicating that the RuO_2/CoO_x interface is the key to improve the OER performance of RuO_2/CoO_x. (c) Comparison of current density (J) of RuO_2/CoO_x and RuO_2 at 1.50 V_{RHE} with identical RuO_2-mass. Note that the performance of CoO_x and RuO_2/CoO_x was provided in Fig. 4a and Supplementary Fig. 26, respectively.

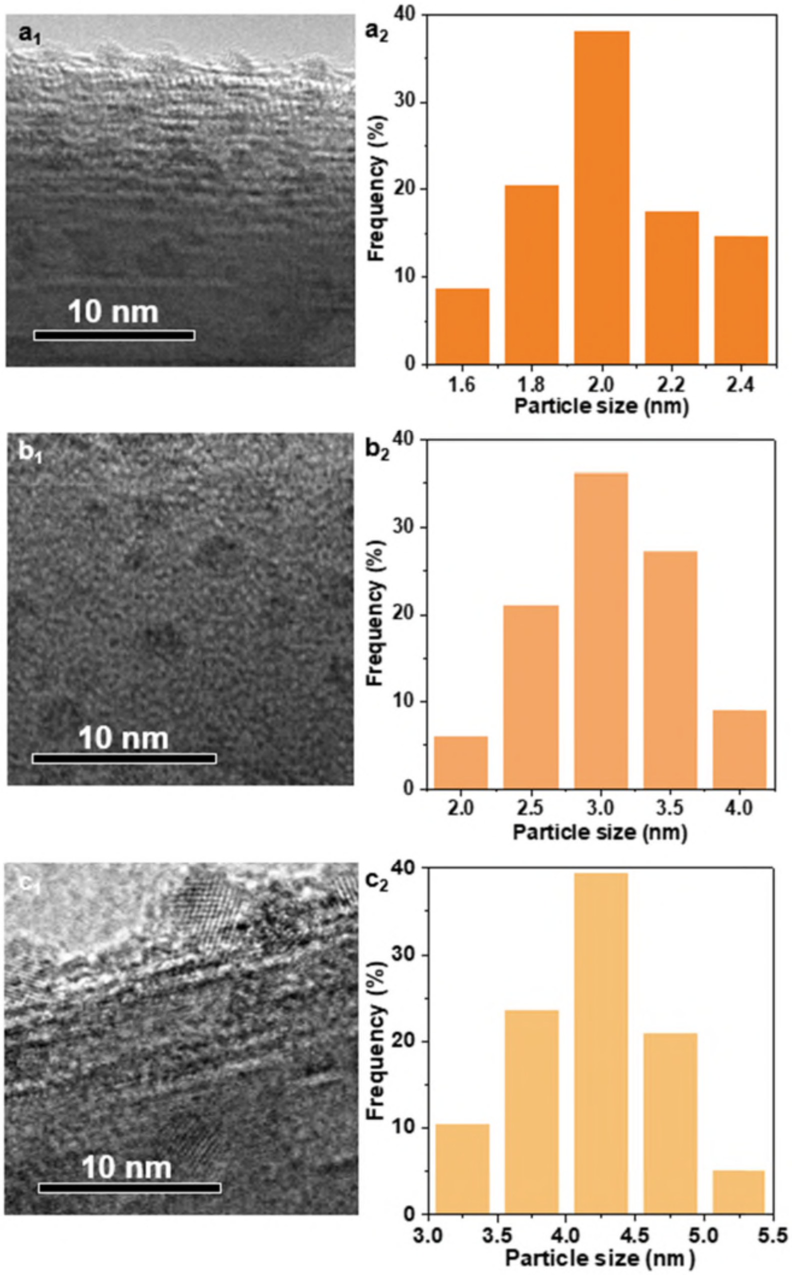

Supplementary Figure 38. TEM images and size distributions of RuO_2/CoO_x catalysts (10 μg_{RuO2}) with different RuO_2 sizes. (a_1) and (a_2) 2 nm. (b_1) and (b_2) 3 nm. (c_1) and (c_2) 4 nm.

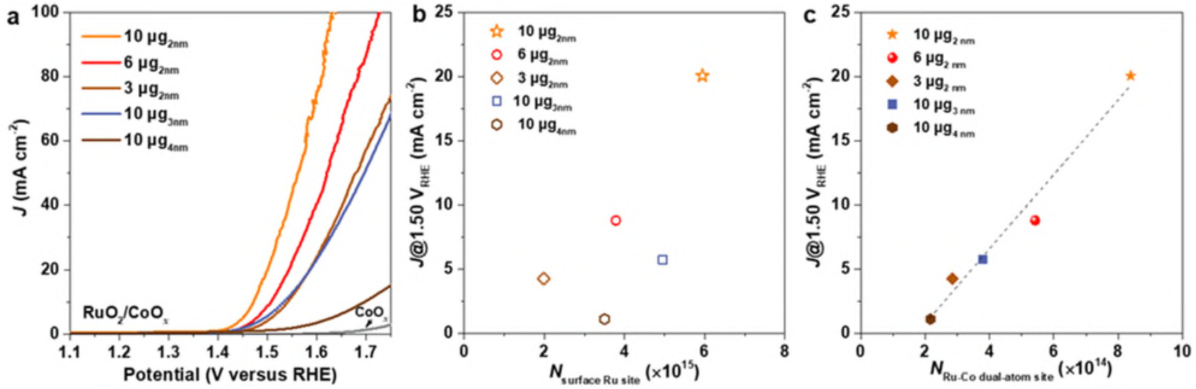

Supplementary Figure 39. (a) Polarization curves of the RuO_2/CoO_x with varied RuO_2 particle size or loading masses and CoO_x in neutral electrolyte. We note that the orange, blue, and dark brown curves represent RuO_2/CoO_x (10 μg_{RuO2}) with RuO_2 particle size of 2, 3 and 4 nm, respectively. (b) and (c) Plot of current density (*J*) for RuO_2/CoO_x at 1.50 V_{RHE} as a function of the estimated number of surface Ru sites and interfacial Ru/Co dual-atom sites, respectively.

Supplementary Tables

Supplementary Table 1. Quantifying the remaining Ru contents in RuO_2/CoO_x and RuO_2 after stability test in 1.0 M PBS by inductively coupled plasma mass spectrometry (ICP-MS).

Catalyst	Treating potential (V_{RHE})	Ru content mass in catalyst (μg)	Remaining Ru content in catalyst after test (wt%)	Remaining Co content in catalyst after test (wt%)
Pristine RuO_2/CoO_x	--	39.90	100.00	100.00
RuO_2/CoO_x-1	1.40	39.81	99.77	99.80
RuO_2/CoO_x-2	1.60	39.76	99.65	99.71
RuO_2/CoO_x-3	1.80	39.71	99.52	99.58
Pristine RuO_2	--	133.13	100.00	--
RuO_2-1	1.40	131.42	98.72	--
RuO_2-2	1.60	123.01	92.40	--
RuO_2-3	1.80	96.07	72.16	--

Supplementary Table 2. Quantifying the remaining Ru contents in RuO_2/CoO_x and RuO_2 after stability test in 1.0 M KOH by ICP-MS.

Catalyst	Treating potential (V_{RHE})	Ru content mass in catalyst (μg)	Remaining Ru content in catalyst after test (wt%)	Remaining Co content in catalyst after test (wt%)
Pristine RuO_2/CoO_x	--	39.90	100.00	100
RuO_2/CoO_x-1	1.40	39.69	99.47	99.63
RuO_2/CoO_x-2	1.60	39.62	99.30	99.39
RuO_2/CoO_x-3	1.80	39.53	99.07	98.97
Pristine RuO_2	--	132.97	100.00	--
RuO_2-1	1.40	131.46	98.87	--
RuO_2-2	1.60	124.06	93.30	--
RuO_2-3	1.80	102.29	76.93	--

Supplementary Table 3. Details of preparing RuO_2/CoO_x[a] for OER test.

Catalyst	m_{RuO_2} (μg cm^{-2})[b]	m_{CoO_x} (mg cm^{-2})[c]	Ru (at%)[d]	$m_{Carbon\ black\text{-}ink}$ (mg cm^{-2})[e]	Total mass (mg cm^{-2})[f]
1	3	0.25	0.7	0.25	0.50
2	6	0.25	1.3	0.25	0.50
3	10	0.25	2.1	0.25	0.50
4	17	0.24	3.9	0.25	0.50
5	30	0.23	7.0	0.25	0.50

[a]loaded 0.05 mg RuO_2/CoO_x and 0.05 mg carbon black on RDE (0.196 cm^2). [b]mass of RuO_2 on per cm^2 RDE, measured by ICP-MS. [c]mass of CoO_x on per cm^2 RDE, $m_{CoOx} = (0.05\ \text{mg-}\ m_{RuO_2})\ /0.196\ \text{cm}^2$. [d]Ru (at%)=$N_{Ru}/(N_{Ru}+N_{Co})$, where N_{Ru} and N_{Co} are the number of Ru and Co in the RuO_2/CoO_x catalyst, respectively. [e]added in the catalyst ink. [f]Total mass = $m_{RuO_2} + m_{CoOx} + m_{Carbon\ black}$.

Supplementary Table 4. Details of preparing reference RuO_2[a] (deposited on carbon black) for OER test.

Catalyst	m_{RuO_2} (μg cm^{-2})[b]	$m_{Carbon\ black}$ (mg cm^{-2})[c]	$m_{Carbon\ black\text{-}ink}$ (mg cm^{-2})[d]	Total mass (mg cm^{-2})[f]
1	23	0.23	0.26	0.50
2	50	0.20	0.26	0.50
3	84	0.17	0.26	0.50
4	126	0.13	0.26	0.50

[a] loaded 0.05 mg RuO_2 (with supporting carbon black) and 0.05 mg carbon black on RDE (0.196 cm^2). [b]mass of RuO_2 on per cm^2 RDE, measured by ICP-MS. [c]mass of carbon black for depositing RuO_2 on per cm^2 RDE, $m_{Carbon\ black} = 0.05\ \text{mg-}\ m_{RuO_2}$. [d]mass of carbon black added in the catalyst ink. [e]Total mass = $m_{RuO_2}+m_{Carbon\ black}+m_{Carbon\ black\text{-}ink}$.

Supplementary Table 5. Performance comparison between RuO_2/CoO_x catalyst and the reported highly active catalysts in neutral solutions.

Catalyst	Electrolyte	Overpotential @ 10 mA cm^{-2} (mV)	Loading (mg cm^{-2})	TOF (s^{-1})	Current density retention after stability test	Reference
**RuO_2/CoO_x**	**1.0 M PBS**	**242**	**0.25**	**1.21@300 mV<br>3.61@400 mV**	**99.8% after 200 h@10 mA cm^{-2}**	**This work**
$RuIrCaO_x$	0.50 M $KHCO_3$	250	0.42	0.36@400 mV	99.3% after 200 h@10 mA cm^{-2}	16
RuO_2@C	1.0 M PBS	269	0.76	--	--	17
PdP_2/C	1.0 M PBS	277	0.28	--	--	18
Ir-NSG[a]	1.0 M PBS	307	0.30	--	97.3% after 4.2 h@1.4 V	19
IrRu@Te[b]	1.0 M PBS	309	0.60	--	--	20
RhCo	1.0 M PBS	310	2.00	--	94.6% after 5 h@1.6 V	21
$N\text{-}Fe_2PO_{5-x}$[c]	1.0 M PBS	315	2.50	--	99.6% after 30 h@50 mA cm^{-2}	22
NiCoFeP	0.50 M $KHCO_3$	330	0.39	--	97.4% after 100 h@10 mA cm^{-2}	23
$Ru\text{-}RuO_2/C_3N_4$	1.0 M PBS	342	0.14	0.03@300 mV	97.5% after 5.5 h @10 mA cm^{-2}	24
$Ir_1\text{-}Co(OH)_2$[d]	1.0 M PBS	373	0.57	--	98.5% after 10 h@10 mA cm^{-2}	25
Co_3O_4	3.0 M KPi	390	0.16	0.01@400 mV	--	26
Co-Pi/Ti[e]	1.0 M PBS	450	0.95	0.07@410 mV	99.4% after 20 h@10 mA cm^{-2}	27

Co-MOF[f]	0.10 M PBS	548@2 mA cm^{-2}	0.71	0.03@400 mV	--	28
$NiFe_2O_4/FeNi_2S_4$	0.20 M PBS	253@1 mA cm^{-2}	0.20	--	--	29
Co_4Mo	0.10 M PBS	490	0.20	0.03@490mV	97.9% after 15 h@10 mA cm^{-2}	30

[a]Ir nanoclusters dispersed on N, S-doped graphene. [b]IrRu intermetallic nanoclusters loaded on tellurium nanoparticle support. [c]Nitrogen-incorporated Fe_2PO_5. [d]Single-atom Ir supported on $Co(OH)_2$ nanosheets. [e]Cobalt phosphate nanoarrays on Ti mesh. [f]Cobalt metal-organic framework.

Supplementary Table 6. Location of *OOH reported in literature.[a]

Catalyst	Location of *OOH (cm^{-1})	Electrolyte	Reference
Pt/C	1212	0.1 M $HClO_4$	31
Ru_1–Pt_3Cu	1212	0.1 M $HClO_4$	32
CoO_x	1054	1.0 M KOH	33
NiFe MOF	1050	0.1 M KOH	34

[a]Note that *OOH bands shift toward lower wavelength direction in alkaline solution compared with those in acidic solution. According to previous work[35], it is ascribed to the fact that the H atom in *OOH can form hydrogen bond with O atom in OH^-, resulting in *OOH to move in the lower wavenumber direction in IR spectra. The location of *OOH on our samples in neutral solution is between the reported values of *OOH in alkaline and acidic solutions.

Supplementary Table 7. Performance comparison between RuO_2/CoO_x catalyst and the reported highly active catalysts in alkaline solutions.

Catalyst	Electrolyte	Overpotential @ 10 mA cm^{-2} (mV)	Loading (mg cm^{-2})	TOF (s^{-1})	Current density retention after stability test	Reference
**RuO_2/CoO_x**	**1.0 M KOH**	**165**	**0.25**	**8.75@300 mV**	97.3% after 20 h@1 A cm^{-2}	**This work**
NiFe-Boride	1.0 M KOH	167	0.39	--	99.7% after 100 h@20 mA cm^{-2}	36
$(Ru-Co)O_x$	1.0 M KOH	171	0.80	0.25@270 mV	98.1% after 10 h@10 mA cm^{-2}	37
Ir/CoNiB	1.0 M KOH	178	--	0.37@300 mV	98.4% after 50 h @ 100 mA cm^{-2}	38
NiO/NiFe LDH[a]	1.0 M KOH	180	0.20	0.71@300 mV	99.8% after 10 h @20 mA cm^{-2}	39
NiFeCu	1.0 M KOH	180	0.45	--	99.7% after 20 h @20 mA cm^{-2}	40
$NiMO_x$/NiMoS	1.0 M KOH	186	--	--	98.6% after 25 h@500 mA cm^{-2}	41
LNF[b]	1.0 M KOH	189	0.23	--	98.7% after 100 h@10 mA cm^{-2}	42
FeCoW	1.0 M KOH	223	0.21	0.46@300 mV	99.8% after 550 h@30 mA cm^{-2}	43
Ru/CoFe-LDH	1.0 M KOH	198	1.0	--	99.5% after 24 h@200 mA cm^{-2}	14
$Mo-Co_9S_8$	1.0 M KOH	200	1.0	--	99.7% after 72 h@10 mA cm^{-2}	44
Ru_1-FeCoNi	1.0 M KOH	205	0.25	--	98% after 48 h@10 mA cm^{-2}	45
$CoV-Fe_{0.28}$[c]	1.0 M KOH	215	0.28	--	96.9% after 40 h@1.55 V	46

$Co_3O_4/Fe_{0.33}Co_{0.66}P$	1.0 M KOH	215@50 mA cm^{-2}	2.5	--	89.4% after 150 h@240 mV	47
NiFeP	1.0 M KOH	218	--	--	98.1% after 30 h@10 mA cm^{-2}	48
Fe-CoP/CoO	1.0 M KOH	219	0.29	--	97.5% after 12 h@10 mA cm^{-2}	49
NiFe LDH[d]	1.0 M KOH	225	0.10	--	--	50
NiFeRu-LDH	1.0 M KOH	225	1.20	--	98.8% after 10 h@10 mA cm^{-2}	51
$CoFeWO_x$	1.0 M KOH	231	0.20	0.54@300 mV	99.4% after 120 h@100 mA cm^{-2}	52
Au_1-NiFe LDH	1.0 M KOH	237	2.0	--	91.2% after 20 h@100 mA cm^{-2}	53
NiFe-LDH	1.0 M NaOH	240	--	--	--	54
$Fe_{0.4}Co_{0.6}Se_2$	1.0 M KOH	270	0.50	1.23@300 mV	98.3% after 24 h@10 mA cm^{-2}	55
Ir_{16}-PdCu	0.1 M KOH	284	0.050	64.1@300 mV	98.6% after 10 h@10 mA cm^{-2}	56
RuO_2	0.5 M KOH	358	0.025	0.53@400 mV	--	57

[a]NiO nanoparticles connecte with NiFe LDH. [b]$FeCl_3$ treated $LaNiO_3$. [c]Cobalt-vanadium-iron (oxy)hydroxide (CoV-$Fe_{0.28}$). [d]NiFe layered double hydroxides (LDHs).

Supplementary References

1. Bratsch, S. G. Standard electrode potentials and temperature coefficients in water at 298.15 K. *J. Phys. Chem. Ref. Data* **18**, 1-21 (1989).
2. Povar, I. & Spinu, O. Ruthenium redox equilibria 3. Pourbaix diagrams for the systems Ru-H_2O and Ru-Cl-H_2O. *J. Electrochem. Sci. Engin.* **6**, 145-153 (2016).
3. Zeng, Z., Chang, K. C, Kubal, J., Markovic, N. M. & Greeley, J. Stabilization of ultrathin (hydroxy)oxide films on transition metal substrates for electrochemical energy conversion. *Nat. Energy* **2**, 1-9 (2017).
4. Suen, N. T., Hung, S. F., Quan,Q., Zhang, N., Xu, Y. J. & Chen, H. M. Electrocatalysis for the oxygen evolution reaction: recent development and future perspectives. *Chem. Soc. Rev.* **46**, 337-365 (2017)..
5. Morgan D. J. Resolving ruthenium: XPS studies of common ruthenium materials. *Surf. Interface Anal.* **47**, 1072-1079 (2015).
6. Dyrek, K. & Sojka, Z. Coordination and dispersion of Co^{2+} ions in CoO-MgO solid solutions. *J. Chem. Soc. Farday Trans. 1* **78**, 3177-3185 (1982).
7. Kim, H. *et al*. Coordination tuning of cobalt phosphates towards efficient water oxidation catalyst. *Nat. Commun.* **6**, 8253 (2015).
8. Gerken, J. B. *et al.* Electrochemical water oxidation with cobalt-based electrocatalysts from pH 0-14: the thermodynamic basis for catalyst structure, stability, and activity. *J. Am. Chem. Soc.* **133**, 14431–14442 (2011).
9. McAlpin, J. G. *et al.* Electronic structure description of a [$Co(III)_3Co(IV)O_4$] cluster: a model for the paramagnetic intermediate in cobalt-catalyzed water oxidation. *J. Am. Chem. Soc.* **133**, 15444–15452 (2011).
10. McAlpin, J. G. *et al*. EPR evidence for Co(IV) species produced during water oxidation at neutral pH. *J. Am. Chem. Soc.* **132**, 6882–6883 (2010).
11. Webster, R. D., Heath, G. A. & Bond, A. M. Voltammetric, EPR and UV-VIS-NIR spectroscopic studies associated with the characterisation of electrochemically generated tris (dithiocarbamato)cobalt(IV) complexes in dichloromethane. *J. Chem. Soc. Dalton Trans.* **21**, 3189–3195 (2001).
12. Rivesta, J. B. & Jain, P. K. Cation exchange on the nanoscale: an emerging technique for new material synthesis, device fabrication, and chemical sensing. *Chem. Soc. Rev.* **42**, 89-96 (2013).
13. Cui, H., Park, J. H. & Park, J. G. Study of ruthenium oxides species on ruthenium chemical mechanical planarization using periodate-based slurry. *J. Electrochem. Soc.* **159**, H335-

H341 (2012).

14. Li, P. *et al.* Boosting oxygen evolution of single-atomic ruthenium through electronic coupling with cobaltiron layered double hydroxides. *Nat. Commun.* **10**, 1711 (2019).

15. Zhao, S. *et al.* Structural transformation of highly active metal-organic framework electrocatalysts during the oxygen evolution reaction. *Nat. Energy* **5**, 881–890 (2020).

16. Zhang, L. S. *et al*. Boosting neutral water oxidation through surface oxygen modulation. *Adv. Mater* **32**, 2002297 (2020).

17. Park, H. S. *et al.* RuO_2 nanocluster as a 4-in-1 electrocatalyst for hydrogen and oxygen electrochemistry. *Nano Energy* **55**, 49–58 (2019).

18. Luo, F. *et al.* Palladium phosphide as a stable and efficient electrocatalystfor overall water splitting. *Angew Chem. Int. Edit.* **57**, 14862 –14867 (2018).

19. Wang, Q. *et al.* Coordination engineering of iridium nanocluster bifunctional electrocatalyst for highly efficient and pH-universal overall water splitting. *Nat. Commun.* **11**, 4246 (2020).

20. Xu, J. *et al.* Strong electronic coupling between ultrafine iridium–ruthenium nanoclusters and conductive, acid-stable tellurium nanoparticle support for efficient and durable oxygen evolution in acidic and neutral media. *ACS Catal.* **10**, 3571−3579 (2020)..

21. Zhao, Y. *et al.* Atomically ultrathin RhCo alloy nanosheet aggregates for efficient water electrolysis in broad pH range. *J. Mater. Chem. A* **7**, 16437–16446 (2019).

22. Wu, Y. *et al.* Orienting active crystal planes of new class lacunaris Fe_2PO_5 polyhedrons for robust water oxidation in alkaline and neutral media. *Adv. Funct. Mater.* **28**, 1801397 (2018)..

23. Zheng, X. L. *et al.* Theory-driven design of high-valence metal sites for water oxidation confirmed using in situ soft X-ray absorption. *Nat. Chem.* **10**, 149-154 (2018).

24. Jiang, B. *et al.* Functionalization of metal oxides with thiocyanate groups: a general strategy for boosting oxygen evolution reaction in neutral media. *Nano Energy* **76**, 105079 (2020).

25. Zhang, Y. K. *et al.* Atomic iridium incorporated in cobalt hydroxide for efficient oxygen evolution catalysis in neutral electrolyte. *Adv. Mater.* **30**, 1707522 (2018).

26. Ni, B. *et al.* Mimic the photosystem II for water oxidation in neutral solution: a case of Co_3O_4. *Adv. Energy Mater.* **8**, 1702313 (2018).

27. Xie, L. *et al.* High-performance electrolytic oxygen evolution in neutral media catalyzed by a cobalt phosphate nanoarray. *Angew Chem. Int. Ed.* **56**, 1064 –1068 (2017).

28. Gutiérrez-Tarriño, S. *et al.* Cobalt metal–organic framework based on layered double nanosheets for enhanced electrocatalytic water oxidation in neutral media. *J. Am. Chem. Soc.* **142**, 19198-19208 (2020).

29. An, L. *et al.* Heterostructure-promoted oxygen electrocatalysis enables rechargeable zinc–air battery with neutral aqueous electrolyte. *J. Am. Chem. Soc.* **140**, 17624−17631 (2018).

30. Xu, Y. T. *et al.* Non-3*d* metal modulation of a cobalt imidazolate framework for excellent electrocatalytic oxygen evolution in neutral media. *Angew Chem. Int. Ed.* **58**, 139–143 (2019).

31. Nayak, S., McPherson, I. J. & Vincent, K. A. Adsorbed intermediates in oxygen reduction on platinum nanoparticles observed by in situ IR spectroscopy. *Angew Chem. Int. Edit.* **130**, 13037–13040 (2018).

32. Yao, Y. *et al.* Engineering the electronic structure of single atom Ru sites via compressive strain boosts acidic water oxidation electrocatalysis. *Nat. Catal.* **2**, 304–313 (2019).

33. Lin, Y. M., Yu, L. H., Song, F. H., Schlögl, R. & Heumann S. In situ identification and time-resolved observation of the interfacial state and reactive intermediates on a cobalt oxide nanocatalyst for the oxygen evolution reaction. *ACS Catal*. **12**, 5345-5355 (2022).

34. Cheng, W. *et al*. Lattice-strained metal–organic-framework arrays for bifunctional oxygen electrocatalysis. *Nat. Energy* **4**, 115-122 (2019).

35. Behera, B. & Das, P. K. Blue- and red-shifting hydrogen bonding: a gas phase FTIR and ab initio study of RR′CO···$DCCl_3$ and RR′S···$DCCl_3$ complexes. *J. Phys. Chem. A* **122**, 4481-4489 (2018).

36. Wang, N. et al. Boride-derived oxygen-evolution catalysts. *Nat. Commun.* **12**, 6089 (2021).

37. Wang, C. & Qi, L. Heterostructured inter-doped ruthenium–cobalt oxide Hollow nanosheet arrays for highly efficient overall water splitting. *Angew Chem. Int. Ed.* **59**, 17219–17224 (2020).

38. Wang, C. *et al.* Engineering lattice oxygen activation of iridium clusters stabilized on amorphous bimetal borides array for oxygen evolution reaction. *Angew Chem. Int. Ed.* **60**, 27126–27134 (2021).

39. Gao, Z. W. *et al.* Engineering NiO/NiFe LDH intersection to bypass scaling relationship for oxygen evolution reaction via dynamic tridimensional adsorption of intermediates. *Adv. Mater.* **31**, 1804769 (2019).

40. Zhang, P. *et al.* Dendritic core-shell nickel-iron-copper metal/metal oxide electrode for efficient electrocatalytic water oxidation. *Nat. Commun.* **9**, 381 (2018).

41. Zhai, P. *et al.* Engineering active sites on hierarchical transition bimetal oxides/sulfides heterostructure array enabling robust overall water splitting. *Nat. Commun.* **11**, 5462 (2020).

42. Chen, G. *et al.* An amorphous nickel–iron-based electrocatalyst with unusual local structures for ultrafast oxygen evolution reaction. *Adv. Mater.* **31**, 1900883 (2019).

43. Zhang, B. *et al.* Homogeneously dispersed multimetal oxygen-evolving catalysts. *Science* **352**, 333-337 (2016).

44. Wang, L. *et al.* Atomically dispersed Mo supported on metallic Co_9S_8 nanoflakes as an advanced noble-metal-free bifunctional water splitting catalyst working in universal pH conditions. *Adv. Energy Mater.* **10**, 1903137 (2020).

45. Hu, Y. *et al.* Single Ru atoms stabilized by hybrid amorphous/ crystalline FeCoNi layered double hydroxide for ultraefficient oxygen evolution. *Adv. Energy Mater.* **11**, 2002816 (2021).

46. Kuang, M. *et al.* Amorphous/crystalline heterostructured cobalt-vanadium iron (oxy)hydroxides for highly efcient oxygen evolution reaction. *Adv. Energy Mater.* **10**, 2002215 (2020).

47. Zhang, X. *et al.* $Co_3O_4/Fe_{0.33}Co_{0.66}P$ interface nanowire for enhancing water oxidation catalysis at high current density. *Adv. Mater.* **30**, 1803551 (2018).

48. Li, R. Q. *et al.* Monolithic electrode integrated of ultrathin NiFeP on 3D strutted graphene for bifunctionally efficient overall water splitting. *Nano Energy* **58**, 870–876 (2019).

49. Hu, X. *et al.* 2D Fe-containing cobalt phosphide/cobalt oxide lateral heterostructure with enhanced activity for oxygen evolution reaction. *Nano Energy* **56**, 109–117 (2019).

50. Dionigi, F. *et al.* In-situ structure and catalytic mechanism of NiFe and CoFe layered double hydroxides during oxygen evolution. *Nat. Commun.* **11**, 2522 (2020).

51. Chen, G. *et al.* Accelerated hydrogen evolution kinetics on NiFe-layered double hydroxide electrocatalysts by tailoring water dissociation active sites. *Adv. Mater.* **30**, 1706279 (2018).

52. Chen, J. *et al.* Octahedral coordinated trivalent cobalt enriched multimetal oxygen-evolution catalysts. *Adv. Energy Mater.* **10**, 2002593 (2020).

53. Zhang, J. *et al.* Single-atom Au/NiFe layered double hydroxide electrocatalyst: probing the origin of activity for oxygen evolution reaction. *J. Am. Chem. Soc.* **140**, 3876−3879 (2018).

54. Luo, J. *et al.* Water photolysis at 12.3% efficiency via perovskite photovoltaics and Earth-abundant catalysts. *Science* **345**, 1593-1596 (2014).

55. Zhang, J. Y. *et al.* Local spin-state tuning of cobalt–iron selenide nanoframes for the boosted oxygen evolution. *Energ. Environ. Sci.* **14**, 365-373 (2021).

56. Qin, Y. *et al.* High valence M-incorporated PdCu nanoparticles (M = Ir, Rh, Ru) for water electrolysis in alkaline solution. *Nano Lett.* **21**, 5774-5781 (2021).

57. Tung, C. W. *et al.* Reversible adapting layer produces robust single-crystal electrocatalyst for oxygen evolution. *Nat. Commun.* **6**, 8106 (2015).
